# Interfacial Interaction‐Engineered Dual Lithiophilic Ultrathin Interlayers for Dendrite‐Suppressed Lithium Metal Textile Anodes

**DOI:** 10.1002/advs.77494

**Published:** 2026-08-29

**Authors:** Chanseok Lee, Donghyeon Nam, Sang Hun Baeck, Boyeon Kim, Yongkwon Song, Jaemin Kim, Daegun Kim, Jun Hyuk Moon, Yoon Jang Chung, Hyeong Jun Kim, Seoin Back, Yongmin Ko, Jinhan Cho

**Affiliations:** ^1^ Department of Chemical and Biological Engineering Korea University Seoul Republic of Korea; ^2^ The George W. Woodruff School of Mechanical Engineering Georgia Institute of Technology Atlanta Georgia USA; ^3^ KU‐KIST Graduate School of Converging Science & Technology Korea University Seoul Republic of Korea; ^4^ Department of Materials Science and Engineering Northwestern University Evanston Illinois USA; ^5^ Department of Chemical and Biomolecular Engineering Sogang University Seoul Republic of Korea; ^6^ School of Chemical Biological and Battery Engineering Gacheon University Seongnam Republic of Korea; ^7^ Department of Integrative Energy Engineering Korea University Seoul Republic of Korea; ^8^ Institute For Multiscale Matter and Systems (IMMS) Ewha Womans University Seoul Republic of Korea; ^9^ Division of Energy & Environmental Technology Materials Research Institute Daegu Gyeongbuk Institute of Science and Technology (DGIST) Daegu Republic of Korea

**Keywords:** functional separators, lithiophilic interlayers, lithium metal anode, lithium metal batteries, textile current collectors

## Abstract

Lithium (Li) metal, known for its exceptional theoretical capacity and low redox potential, represents a promising anode material for next‐generation Li‐based batteries. However, its practical implementation is often compromised by uncontrolled dendritic Li growth during cycling, leading to safety hazards and accelerated degradation. In this study, we present a novel dual‐interface engineering strategy aimed at mitigating Li dendrite formation through the incorporation of ultrathin lithiophilic interlayers on both the electrode (∼44 nm) and separator (∼2 nm). We utilize a Ni‐electroplated textile as a conductive host, onto which lithiophilic Ag nanoparticles and amine‐based molecular linkers are uniformly assembled via a ligand‐exchange‐mediated approach, resulting in an ultrathin lithiophilic interlayer. Simultaneously, the separator is modified through a hydrogen‐bonding‐driven assembly of NH_2_‐functionalized poly(ethylene imine) and COOH‐functionalized poly(acrylic acid), forming a ∼2 nm interlayer that redistributes Li^+^ flux. This synergistic interfacial design enables symmetric cells to operate stably for over 2200 h at 1 mA cm^−^^2^/1 mAh cm^−^^2^, while lithium iron phosphate‐based full cells retain approximately 72% capacity after 5000 cycles at 1 C. Our findings underscore dual‐interface engineering as an effective and scalable approach for the development of safe and durable Li metal batteries.

## Graphical abstract

Ultrathin lithiophilic interlayers engineered on a 3D textile electrode and separator cooperatively regulate interfacial Li nucleation and Li^+^ transport, leading to homogeneous Li plating and mitigated dendrite formation. The resulting dual‐interfacial design provides long‐term cycling stability, improved full‐cell durability, and a scalable pathway toward practical high‐energy Li metal batteries.

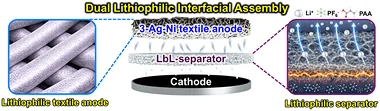

## 1 Introduction

Lithium (Li) metal is considered the leading anode material for next‐generation rechargeable batteries due to its high theoretical capacity (3860 mAh g^−^^1^), low redox potential (−3.04 V vs. SHE), and low density [1, 2, 3, 4]. However, practical use of lithium metal anodes (LMAs) is hindered by interrelated instabilities during cycling, including high reactivity, significant volumetric changes, and fragile solid–electrolyte interphase (SEI) [5, 6, 7, 8]. These issues lead to uncontrolled dendritic growth and the accumulation of “dead lithium,” posing safety risks and accelerating capacity loss [9, 10, 11, 12, 13, 14]. Chen et al. clarified that the formation of dead Li^0^ during Li stripping is governed by electron transfer, Li^0^‐to‐Li^+^ oxidation kinetics, and Li^+^ diffusion through the SEI [15]. Abdollahifar and Paolella further summarized that electrically isolated Li fragments trapped by the SEI become electrochemically inactive, leading to capacity loss, increased interfacial resistance, heat generation, and aggravated dendrite‐related safety concerns [16]. The core of these failures lies in the uneven local current density, which causes nonuniform Li nucleation and growth [17, 18, 19, 20].

To mitigate these issues, significant efforts have focused on engineering lithiophilic interfaces on 2D host electrodes [21, 22]. Among inorganic lithiophilic agents, silver (Ag) and gold (Au) particles larger than 50 nm are particularly effective in promoting uniform Li nucleation by lowering the energy barrier and enhancing Li^+^ affinity [23, 24, 25, 26]. Ag, in particular, forms Li–Ag solid‐solution interfaces that reduce nucleation overpotential and facilitate homogeneous deposition [27, 28]. However, conventional coating approaches typically rely on slurry‐cast layers with insulating polymer binders, resulting in thick coatings that compromise energy density and impede ion transport [29, 30]. An ideal design would instead employ ultrathin lithiophilic layers densely packed with active binding sites and free of inactive binders—an outcome that remains elusive with conventional fabrication techniques.

3D porous host electrodes, such as nickel (Ni) foams, offer a partial solution to these challenges, as their expanded surface area effectively reduces local current density [31, 32]. However, when fabricated via conventional slurry casting, their porous architecture often impedes uniform coating of lithiophilic components, while their relatively high mass further diminishes electrode‐level energy density [33, 34].

A particularly promising yet underexplored category of 3D hosts is fiber‐based textile substrates—either natural or synthetic (such as cotton or polyester)—which offer significantly lower mass densities (0.12–0.16 g cm^−^^3^) compared to conventional Ni foams (1.0–1.5 g cm^−^^3^). Nonetheless, their inherent electrical insulation and complex morphology have severely limited their application as host electrodes. These limitations become particularly critical under limited‐Li and practically demanding cell conditions, where highly reversible Li plating is required [35, 36]. Moreover, increasing the surface area or modifying the microtexture alone does not necessarily ensure stable Li deposition [37]. Accordingly, bridging this gap requires not only enhancing electrical conductivity but also developing a conformal, binder‐free lithiophilic coating capable of regulating interfacial lithiophilicity and uniformly infiltrating the entire 3D fibrous architecture [38]—an objective that remains challenging for recent LMA studies [39, 40]. Beyond such host electrode engineering, effective control of Li deposition demands a holistic approach in which separator engineering serves as a valuable complementary strategy [41, 42, 43, 44]. An ideal separator should enable uniform Li^+^ transport while preventing electron conduction and dendrite penetration. However, conventional separators often have limited functionality for regulating Li^+^ flux and suppressing anion migration, leading to interfacial heterogeneity during cycling. Incorporating lithiophilic interlayers in separators has been explored to stabilize Li^+^ flux and reduce dendritic growth [45, 46, 47, 48]. Yet, like electrode strategies, current separator methods typically use slurry‐cast, binder‐containing layers that increase structural complexity and degrade ion transport and reaction kinetics [49, 50, 51, 52]. Consequently, both electrode and separator strategies share the challenge of needing conformal, ultrathin, binder‐free lithiophilic modifications that current technologies cannot achieve simultaneously. Even with successful homogeneous nucleation on the electrode surface, nonuniform Li^+^ flux from the separator can still cause localized deposition. Conversely, regulating ion flux from the separator alone cannot fully prevent dendrite formation when the electrode surface has energetically heterogeneous nucleation sites.

These considerations lead to a unified design principle: since Li deposition is influenced by the interconnected processes of ion transport and interfacial nucleation at the electrode–separator interface [53, 54, 55, 56], simultaneous regulation of both surfaces provides a more effective approach than optimizing either in isolation. A dual‐interface strategy could align lithiophilic guidance from both sides, suppressing dendrite formation at the anode while homogenizing Li^+^ flux at the separator, thereby addressing the root causes of instability cohesively. In practice, however, such an approach remains a formidable challenge: existing methods are inherently constrained by thick, nonconformal coatings and insufficiently robust interfacial interactions, leaving the transformative potential of dual‐interface regulation largely unrealized.

**SCHEME 1:**
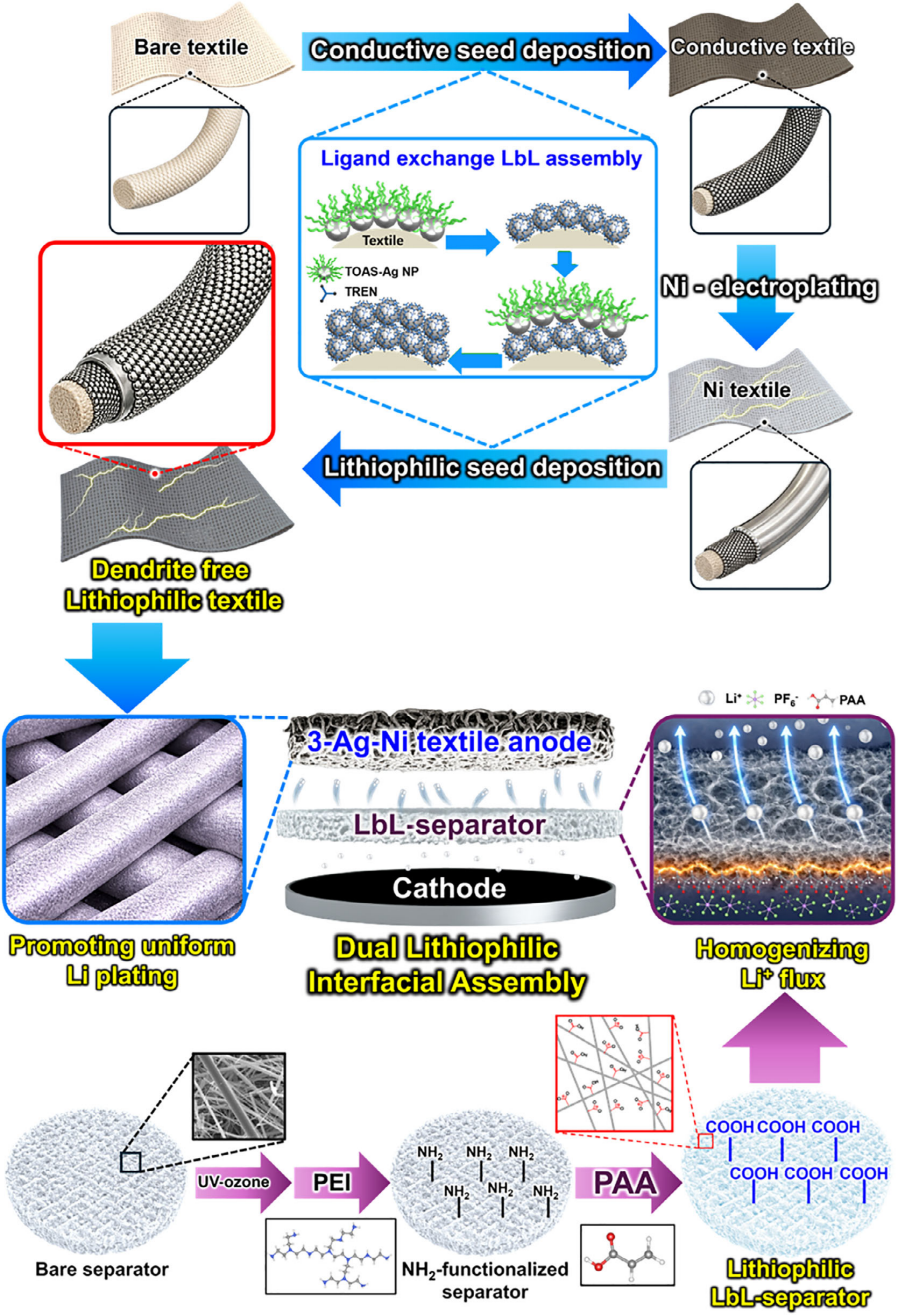
Schematic illustration for the preparation of [TREN/Ag NP]*_n_*‐deposited Ni textile and [PAA/PEI]*_n_*‐deposited separator using a LbL assembly.

Herein, we present a fundamentally novel dual‐interface engineering strategy that overcomes these longstanding limitations, enabling dendrite‐free lithium metal textile anodes through the integration of ultrathin, binder‐free, and highly lithiophilic interlayers on both the 3D textile host electrode and the separator (Scheme 1). Distinct from previously reported approaches, our strategy simultaneously establishes conformal and mechanically robust interphases at two critical junctions—the electrode–interlayer and separator–interlayer interfaces—while maintaining a total interlayer thickness below 50 nm. These ultrathin hybrid interlayers are constructed via an interfacial interaction–driven layer‐by‐layer (LbL) assembly [57, 58] process based on sequential solution dipping, which synergistically combines ligand‐exchange‐mediated covalent bonding (for electrode modification) with hydrogen‐bonding interactions (for separator modification), thereby enabling precise nanoscale control over thickness and composition across complex 3D architectures.

The synergistic incorporation of these dual lithiophilic interlayers significantly enhances ionic conductivity and Li^+^ transference number, while simultaneously reducing nucleation overpotential and charge transfer resistance, compared to systems employing only a single interlayer. As a result, the integrated configuration— 3‐Ag‐Ni textile | LbL‐separator | 3‐Ag‐Ni textile—delivers stable Li plating/stripping over 2,200 h at 1 mA cm^−^^2^/1 mAh cm^−^^2^ with a low overpotential of 33 mV. Full cells paired with LiFePO_4_ (LFP) cathodes at an LFP loading of 0.91 mg cm^−2^ demonstrate outstanding long‐term stability, retaining 90% and 72% of initial capacity after 2000 and 5000 cycles. To further assess the applicability of the dual‐interface modification under more practically relevant cathode loading and limited Li inventory, the LFP loading was increased up to 7.24 mg cm^−2^ while the N/P ratio was reduced to 1.4. Under these conditions, the full cells achieved a high LFP‐normalized specific energy of 530.1 Wh kg^−1^, calculated based on the mass of LFP active material. Notably, these performances were achieved with only a small fraction of the Li inventory typical of conventional Li chips—less than one‐fifth under standard full cell conditions and ∼3.3% under practical high‐loading/low‐N/P conditions. These results collectively validate dual‐interface engineering as a scalable, materials‐efficient platform for realizing safe and high‐energy lithium metal batteries.

## 2 Results and Discussion

### 2.1 Fabrication of [TREN/Ag NP]*_n_* Multilayer‐Assembled Ni Textile

The quality of the Li plating process is strongly influenced by the surface characteristics of the host electrode materials, including their geometric structure (uniformity and large surface area), chemical composition (lithiophilicity), and electrical conductivity. To this end, we designed ultrathin and uniform lithiophilic multilayers on 3D‐structured conducting textiles as a host material for Li plating, employing a well‐defined LbL assembly of lithiophilic nanomaterials.

We first prepared a highly conductive textile current collector by assembling ∼8 nm TOAS‐stabilized Ag NPs and TREN onto a highly porous polyester textile through covalent interactions between the Ag NP surface and the NH_2_ groups of TREN (Figure S1; Detailed Experimental Section). During this LbL assembly, the inactive bulky TOAS ligands initially attached to the Ag NP surface were almost completely replaced by the TREN molecular linkers with an extremely low molecular weight (M_w_ ∼146). This process significantly reduces the interparticle distance between adjacent Ag NPs, forming a well‐connected electronic network with minimal pore volume. The effective replacement of the lithiophobic TOAS ligands suggests that the resulting LbL multilayers are entirely composed of lithiophilic components (i.e., Ag NPs and NH_2_ moieties of TREN linkers), with consistent adsorption per bilayer.

**FIGURE 1:**
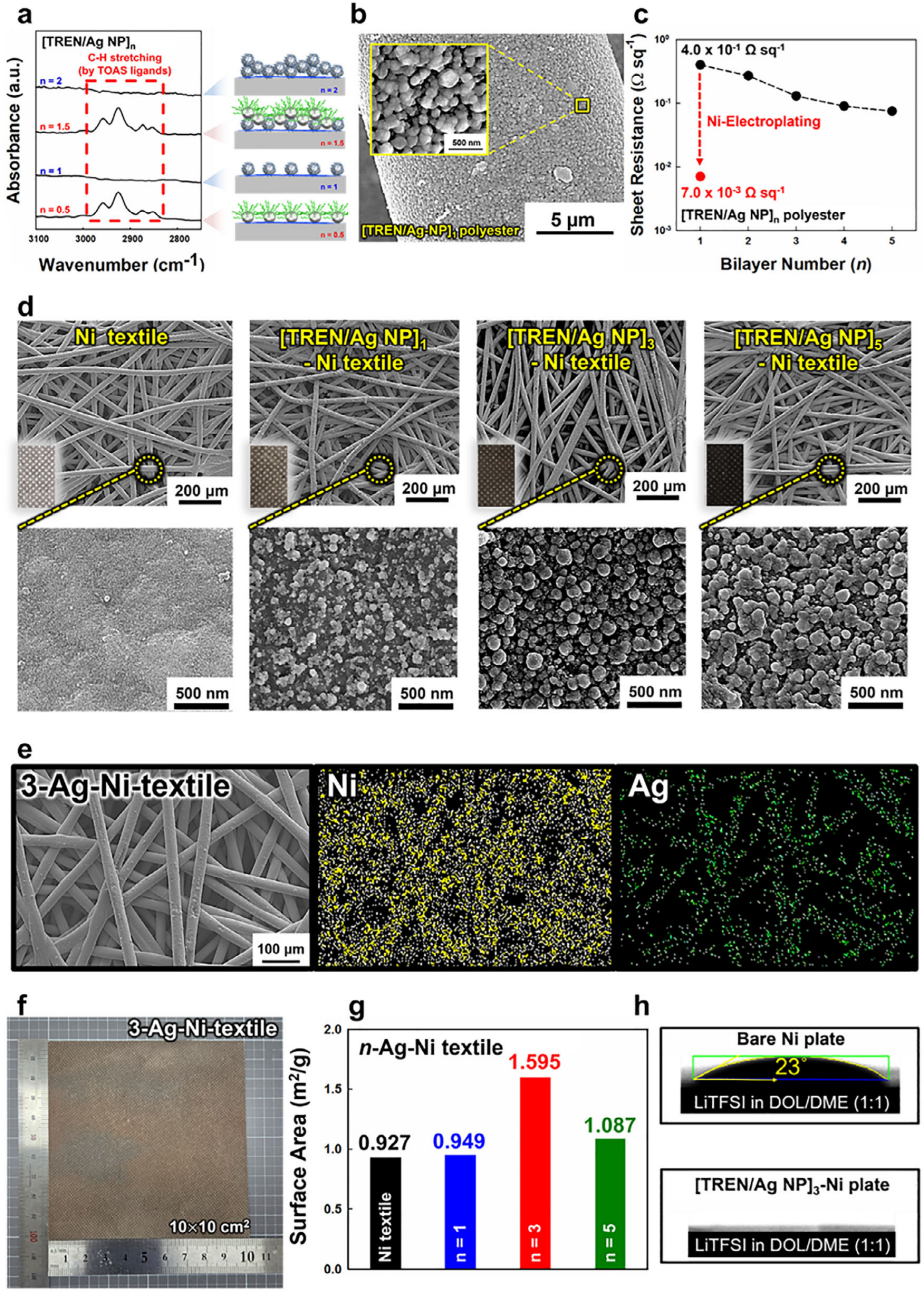
Fabrication of [TREN/Ag NP]*_n_* Multilayer‐Assembled Ni Textile. (a) FTIR spectra and corresponding schematic illustrations of the LbL assembly of [TREN/Ag NP]*_n_* multilayers as a function of bilayer number (*n*). (b) FE‐SEM images of [TREN/Ag NP]_1_‐coated polyester fiber. (c) Change in sheet resistance (Ω sq^−1^) of [TREN/Ag NP]*_n_*‐coated polyester textiles and Ni electroplating/[TREN/Ag NP]_1_/polyester textile. (d) FE‐SEM images of bare Ni textile and *n*‐Ag‐Ni textiles. The inset shows the digital images of the respective textiles. (e) EDS elemental mapping images of 3‐Ag‐Ni textile. (f) Digital photograph of a 10 × 10 cm^2^ 3‐Ag‐Ni textile electrode, demonstrating the practical scalability of the solution‐processable LbL assembly process on a large‐area 3D textile substrate. (g) Surface area of bare Ni textile and *n*‐Ag‐Ni textiles. (h) Electrolyte contact angle measurements for bare Ni plate and [TREN/Ag NP]_3_‐coated Ni plate.

This ligand‐exchange‐induced adsorption behavior of LbL‐assembled [TREN/Ag NP]*_n_* multilayers with increasing bilayer number was systematically analyzed by Fourier transform infrared (FTIR) spectroscopy (Figure 1a). Upon immersing the NH_2_‐functionalized substrate into a TOAS‐Ag NP‐dispersed toluene solution, the FTIR absorption peaks indicative of C–H stretching (at 2,850–2,950 cm^−1^) from the bulky alkyl chains of TOAS ligands were observed, confirming the deposition of the TOAS‐Ag NP layer (*n* = 0.5). These characteristic peaks disappeared completely after immersing the TOAS‐Ag NP‐coated substrate in a TREN‐dissolved ethanol solution (*n* = 1), suggesting the complete removal of loosely bound TOAS ligands from the Ag NP surface. Given the higher affinity of the NH_2_ groups of TREN molecules for the metal surface—due to their lone pairs of electrons—compared to the ammonium groups of TOAS ligands, these FTIR spectroscopic results support the direct adsorption of TREN onto the Ag NP surface through an effective ligand‐exchange reaction. This trend is consistently observed with increasing bilayer number, confirming the successful LbL assembly of [TREN/Ag NP]*_n_* multilayers. Consequently, the organic linker connecting adjacent Ag NPs consisted solely of the small TREN molecule. This reduced interparticle distance was further supported by UV–vis spectroscopy (Figure S2), in which the characteristic surface plasmon resonance peak of TOAS‐Ag NPs in toluene gradually disappeared after assembly, indicating a marked decrease in the separation distance between adjacent Ag NPs.

The [TREN/Ag NP]_1_ bilayer deposited onto polyester textile served as a conductive seed layer and exhibited a low sheet resistance of 0.4 Ω sq^−1^, resulting in densely packed arrays of Ag NPs (Figure 1b,c). The X‐ray diffraction (XRD) pattern of the [TREN/Ag NP]_1_‐coated polyester displayed distinct diffraction peaks at 38.1°, 44.6°, 64.7°, 77.6°, and 81.5°, which correspond well to the crystalline phases of Ag (Figure S3).

Building upon this conductive seed layer, we further enhanced the electrical conductivity of the textile host electrode by electroplating nickel (Ni) onto the [TREN/Ag NP]_1_‐coated polyester. This process reduced the sheet resistance dramatically to 7 × 10^−3^ Ω sq^−1^ after a single electroplating step, proving far more efficient than repeated Ag NP multilayer deposition for constructing a highly conductive textile current collector (Figure 1c). Importantly, the resulting Ni‐electroplated textile (hereafter denoted as “Ni textile”) formed a smooth and continuous metallic layer on individual fibers while preserving the highly porous structure of the pristine polyester without blockage by metal aggregates.

After constructing the conductive Ni textile (mass density: ∼0.7 g cm^−3^), the surface was briefly primed with ultrathin PEI/PAA layers to promote interfacial adhesion, followed by the redeposition of [TREN/Ag NP]_n_ multilayers (n = 1–5) as lithiophilic nanoparticle layers. To quantitatively evaluate the bilayer‐dependent adsorption behavior and lateral packing characteristics of these lithiophilic [TREN/Ag NP]_n_ coatings, quartz crystal microbalance (QCM) measurements were performed (Figure S4). The frequency shifts (−Δ*f*) and corresponding mass changes associated with the sequential adsorption of TREN and Ag NPs were monitored as a function of layer number. The mass uptake was calculated from the measured frequency changes using the Sauerbrey equation, under the assumption that the deposited layers are rigid, uniformly distributed, and sufficiently thin, where Δ*m*, Δ*f*, *C and n* denote the mass change, frequency change, Sauerbrey constant, and harmonic number, respectively.

(1) SauerbreyequationΔm=−CΔf/n

Notably, the amount of adsorbed Ag NPs was found to be strongly governed by the underlying TREN sublayer, irrespective of whether the substrate was a quartz crystal or a polyester textile. As a result, the deposition of Ag NPs onto the TREN layer induced −Δ*f* of 86.4 ± 7.5 Hz, corresponding to a mass loading of ∼1.53 µg cm^−^^2^ per bilayer. Based on an average Ag NP diameter of ∼8 nm, the areal number density of the optimized coating layer was estimated to be approximately 1.63 × 10^12^ cm^−^^2^, corresponding to an estimated effective packing density of ∼82% in the lateral dimension. It is noteworthy that conventional electrostatic assembly of negatively charged metal nanoparticles onto positively charged organic layers in aqueous media typically suffers from limited packing density (<30%) because of strong interparticle electrostatic repulsion [59, 60]. In comparison, the ligand‐exchange‐driven assembly employed here, conducted in organic media, likely facilitates denser nanoparticle packing, underscoring the effectiveness of this interfacial engineering strategy. Atomic MD simulations of the [TREN/Ag NP] interlayer reported by Song et al. further showed that the separation distance between adjacent Ag NPs was estimated to be approximately 6 Å [61]. This molecular‐scale interparticle spacing, together with the negligibly small mass of each TREN layer, indicates that TREN is incorporated as a confined molecular linker between neighboring Ag NPs rather than as a substantial NH_2_‐rich phase. Therefore, the amount of TREN present within the ultrathin [TREN/Ag NP] interlayer is expected to be intrinsically limited.

Despite this additional multilayer growth, the redeposited [TREN/Ag NP]_n_ layers maintained a minimal thickness of only a few tens of nanometers, remaining below 80 nm, while effectively increasing the surface area through their nanoporous architecture (Figure S5). These characteristics contribute to the mitigation of current localization and the reduction of nucleation overpotential, thereby promoting more uniform Li plating. Figure 1d shows the change in surface morphologies of [TREN/Ag NP]*_n_* multilayer‐coated Ni textiles (hereafter denoted as ‘*n*‐Ag‐Ni textile’) with increasing bilayer number (*n*) from 0 to 5, which was confirmed by FE‐SEM. While all multilayer‐coated Ni textiles maintained their highly porous structure, an increase in bilayer numbers led to the formation of larger Ag NPs through the coalescence of smaller TOAS‐Ag NPs, an effect similar to Ostwald ripening, wherein smaller particles combine to form larger entities. As already mentioned, the ligand exchange from bulky pristine TOAS ligands to significantly smaller TREN molecules during LbL assembly reduces the separation distance between adjacent Ag NPs. This decrease in separation distance, along with a low cohesive energy of 2.95 eV per Ag atom, facilitated mutual atom diffusion within the Ag NPs, particularly among those separated by a single TREN molecule. As shown in Figure 1e, the energy dispersive spectroscopy (EDS) elemental mapping confirmed the homogeneous distribution of Ni and Ag throughout the entire 3‐Ag‐Ni textile surface, which was further supported by XRD analysis showing distinct characteristic peaks at 38.1°, 44.6°, 51.9°, 64.7° and 76.8° corresponding to the crystalline bulk structures of Ni and Ag (Figure S6).

Beyond this local compositional uniformity, the solution‐processable LbL assembly could be readily extended to a 10 × 10 cm^2^ textile substrate, yielding a large‐area 3‐Ag‐Ni textile electrode with a uniform macroscopic appearance (Figure 1f; Figure S7). This result highlights the practical scalability of the fabrication process and demonstrates its capability to uniformly integrate lithiophilic active components over large‐area and geometrically complex 3D textile electrodes.

An additional area of interest is the ability of the lithiophilic Ag NP assembly to increase the surface area of the Ni‐textile. To investigate this further, we conducted Brunauer‐Emmett‐Teller (BET) surface area measurements to evaluate the surface area effect of the *n*‐Ag‐Ni textile (Figure 1g; Figure S8). Initially, the pristine Ni textile had a surface area of approximately 0.927 m^2^ g^−1^, which modestly increased to 0.949 m^2^ g^−1^ following the deposition of 1 bilayer (i.e., 1‐Ag‐Ni textile). Notably, with the addition of 3 bilayers, the surface area significantly expanded to 1.595 m^2^ g^−1^, suggesting the formation of an optimal nanoporous structure induced by the fused Ag NPs, thus maximizing surface accessibility while preserving the uniformity of the overall microporous structure. However, increasing the bilayer number (*n*) up to 5 resulted in a decrease in surface area to 1.087 m^2^ g^−1^, likely due to the excessive fusion of Ag NPs, which reduced the accessible surface area. Additionally, the average pore size of the *n*‐Ag‐Ni textiles decreased gradually with increasing bilayer number. This can be attributed to the progressive deposition and partial fusion of Ag NPs along the Ni‐textile framework, which narrows the pore channels by partially occupying the original pore volume. These findings demonstrate that our approach effectively optimizes the surface area of the textile anode by leveraging both the nanoporous structure of the lithiophilic Ag NP multilayers and the macroporous structure of the textile current collector, thereby facilitating uniform current flux at the electrode/electrolyte interface.

Additionally, to evaluate the electrolyte wettability associated with the lithiophilic surface chemistry arising from the LbL‐assembled [TREN/Ag NP]*_n_* multilayers, contact angle measurements were performed using a flat 2D Ni plate. The [TREN/Ag NP]_3_‐coated Ni plate exhibited superior electrolyte wettability compared to the bare Ni plate (Figure 1h). Specifically, the effective exchange of native TOAS ligands on Ag NPs with TREN during the LbL assembly process resulted in an electrolyte‐wettable surface, producing an extremely low contact angle of the electrolyte (1 M lithium bis(trifluoromethanesulfonyl)imide (LiTFSI) in a 1:1 (v/v) mixture of 1,2‐dimethoxyethane (DME) and 1,3‐dioxolane (DOL) containing 5 wt % lithium nitrate (LiNO_3_)). This excellent electrolyte affinity minimizes interfacial impedance, thereby enhancing energy efficiency.

### 2.2 Electrochemical Properties of [TREN/Ag NP]*_n_*‐Coated Ni Textile Anode

**FIGURE 2:**
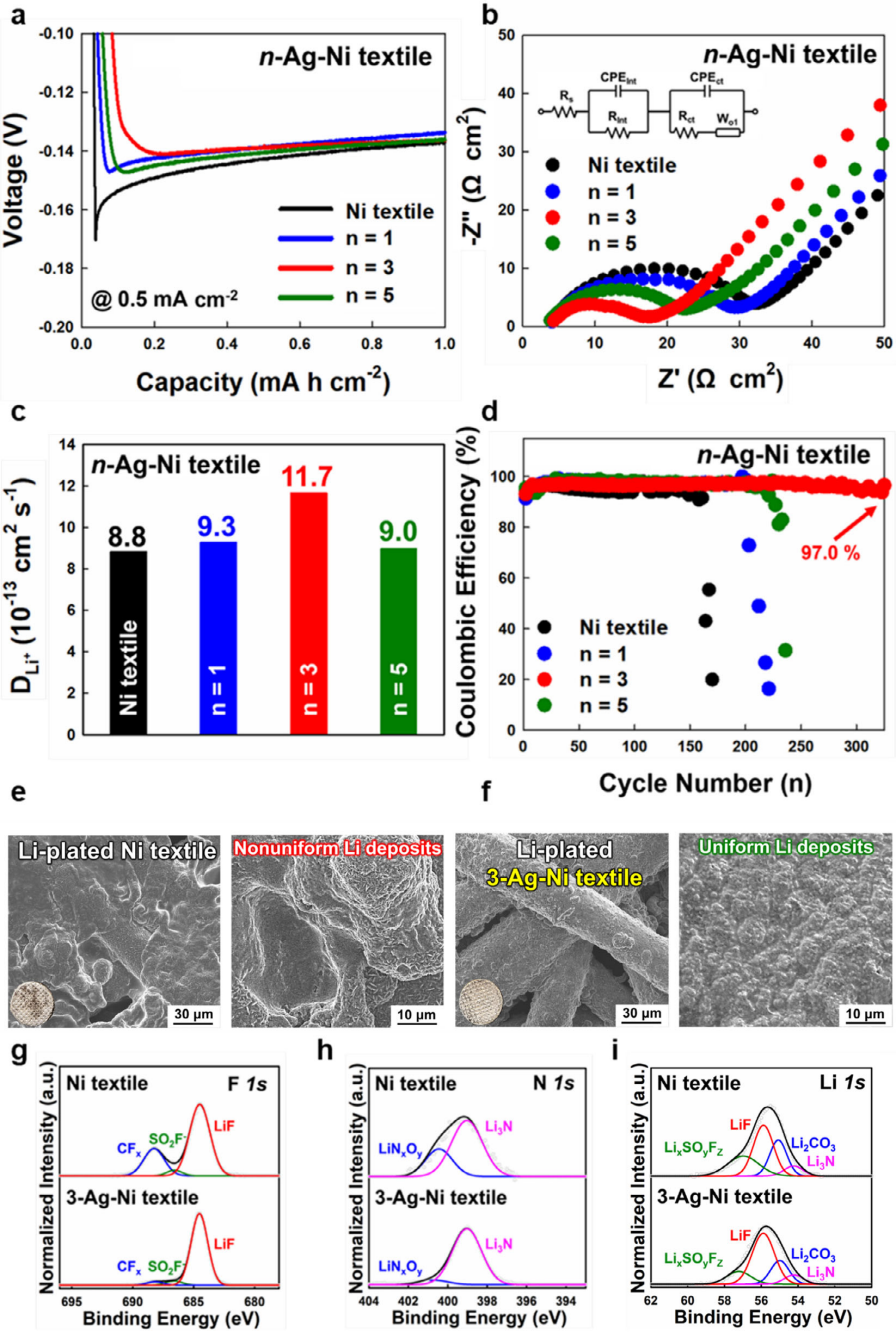
Electrochemical Properties of [TREN/Ag NP]*_n_*‐coated Ni Textile Anode. (a) Voltage (V) vs. capacity (mAh cm^−2^) profiles of *n*‐Ag‐Ni textile‐based half‐cells during Li nucleation at 0.5 mA cm^−2^ and 1 mAh cm^−^^2^. (b) Nyquist plots for *n*‐Ag‐Ni textiles and (c) the corresponding ion diffusion coefficients (*D*_Li_^+^). (d) Coulombic efficiency (%) of *n*‐Ag‐Ni textile‐based half‐cells during repetitive Li plating/stripping cycles at 1 mA cm^−2^ and 1 mAh cm^−2^. FE‐SEM images for the surface morphologies of (e) bare Ni textile and (f) 3‐Ag‐Ni textile in half‐cell configurations after Li plating at 0.5 mA cm^−^^2^ to a capacity of 10 mAh cm^−^^2^. The insets show digital photographs of the Li‐plated textiles. Deconvoluted XPS spectra of bare Ni textile and 3‐Ag‐Ni textile collected after 10 CV cycles of Li plating/stripping: (g) F 1s, (h) N 1s, and (i) Li 1s.

To systematically assess the influence of lithiophilic [TREN/Ag NP]*_n_* multilayers on Li nucleation and growth behavior, we prepared Li | separator | *n*‐Ag‐Ni textile half‐cells with different bilayer numbers (*n*). During Li plating at a current density of 0.5 mA cm^−^^2^ and a capacity of 1 mAh cm^−^^2^, the *n*‐Ag‐Ni textiles exhibited significantly lower nucleation overpotentials than the bare Ni textile and the nonporous Ni foil (Figure 2a; Figure S9). Specifically, increasing the bilayer number (*n*) of *n*‐Ag‐Ni textiles from 0 (bare Ni textile) to 5 resulted in a notable reduction in overpotential values from approximately 33.1 mV (*n* = 0) to 11.2 mV (*n* = 1), 4.9 mV (*n* = 3), and 8.8 mV (*n* = 5). Notably, the 3‐Ag‐Ni textile‐based half‐cell exhibited the lowest nucleation overpotential, indicating a smaller driving force required for the nucleation process. This trend closely followed the variations in effective surface area with different bilayer numbers (see Figure 1g), suggesting that the enlarged electrochemically active surface plays a critical role in reducing local current density and providing abundant Li nucleation sites.

Electrochemical impedance spectroscopy (EIS) was conducted to further investigate the internal resistance and Li^+^ transport kinetics at the interface of the *n*‐Ag‐Ni textile‐based system during Li plating (Figure 2b; Figure S10). The charge‐transfer resistance (*R*_ct_) of the *n*‐Ag‐Ni textile‐based half‐cells decreased markedly with increasing bilayer number (*n*), from 29.9 Ω cm^2^ for the bare Ni textile (*n*  =  0) to 26.3 Ω cm^2^ (*n*  =  1) and 12.1 Ω cm^2^ (*n*  =  3), followed by a slight increase to 19.2 Ω cm^2^ at *n*  =  5. To further evaluate the Li^+^ transport behavior, the Li^+^ diffusion coefficient (D_Li+_) was estimated from the low‐frequency Warburg region based on the linear relationship between *Z*′ and ω^−1/2^. Consistent with the *R*_ct_ trend, the Li^+^ diffusion coefficient (D_Li+_), estimated from the low‐frequency Warburg region, increased from 8.82 × 10^−13^ cm^2^ s^−1^ for the bare Ni textile to 9.29 × 10^−13^ cm^2^ s^−1^ for the 1‐Ag‐Ni textile and reached a maximum of 1.17 × 10^−12^ cm^2^ s^−1^ for the 3‐Ag‐Ni textile, followed by a slight decrease to 8.99 × 10^−13^ cm^2^ s^−1^ for the 5‐Ag‐Ni textile (Figure 2c). These results indicate that the optimized bilayer architecture facilitates Li^+^ transport while reducing interfacial resistance, thereby promoting more efficient Li nucleation and uniform plating.

Building on these electrochemical observations, we evaluated the Coulombic efficiency to assess the long‐term cycling stability of the *n*‐Ag‐Ni textile anodes (Figure 2d; Figure S11). Although the larger surface area of the bare Ni textile moderately improved interfacial stability during Li plating/stripping relative to the nonporous Ni foil by alleviating local current density concentration, a rapid CE decline was observed after ∼150 cycles. This behavior primarily arises from the absence of intrinsic lithiophilic sites and effective interfacial regulation, leading to nonuniform Li nucleation and the promotion of irreversible parasitic side reactions. The introduction of a single Ag NP layer (1‐Ag‐Ni textile) delayed the onset of CE degradation to ∼200 cycles, indicating modest improvements in interfacial uniformity. Further increasing the bilayer number to *n* = 3 resulted in the most stable cycling performance. The 3‐Ag‐Ni textile maintained an average CE of 97.0% without noticeable degradation over more than 320 cycles, attributable to the well‐regulated Li^+^ flux and the suppression of parasitic reactions enabled by the uniformly distributed Ag NP array. In contrast, the 5‐Ag‐Ni textile displayed earlier CE deterioration, beginning around 230 cycles, followed by a rapid decline. This instability arises from excessive Ag NP deposition that reduces the effective surface area and disrupts uniform Li plating. Collectively, these results demonstrate that the 3‐Ag‐Ni textile achieves an optimal balance of lithiophilicity, interfacial stability, and long‐term cycling performance, establishing it as the most effective architecture for high‐performance LMAs.

To establish a direct correlation between electrochemical stability and Li deposition morphology, ex situ FE‐SEM imaging was conducted after Li plating at 0.5 mA cm^−^^2^ to a capacity of 10 mAh cm^−^^2^ (Figure 2e,f). The bare Ni textile exhibited relatively rough and non‐uniform Li deposition with locally protrusive features, reflecting the comparatively lower lithiophilicity of the electroplated Ni surface relative to the 3‐Ag‐Ni textile and the resulting inhomogeneous Li nucleation behavior. It should be noted, however, that the electroplated Ni layer used to prepare the bare Ni textile contains a non‐negligible amount of lithiophilic metal oxides (i.e., Ni(OH)_2_ and NiO) in addition to metallic Ni, which render it slightly more lithiophilic compared with conventional Ni foil or Ni plate (Figure S12; Figure S13). Such interfacial heterogeneity of the bare Ni textile can promote uneven Li growth during continued cycling, thereby increasing the risk of capacity degradation and unstable cell operation (Figure 2e).

In contrast, the 3‐Ag‐Ni textile showed markedly more uniform Li deposition morphology, evolving toward a dense mossy structure during electrochemical operation (Figure 2f). This improvement arises from the enhanced lithiophilicity and uniform Ag NP arrangement provided by the [TREN/Ag NP]_n_ multilayer assembly, which regulates Li^+^ flux and suppresses the development of localized high–surface–energy protrusions. The more uniform and compact Li deposition observed on the 3‐Ag‐Ni electrode further confirms that the optimized Ag NP configuration effectively homogenizes Li nucleation and growth, thereby enabling more stable and reversible Li metal cycling.

To gain molecular‐level insight into this dendrite‐to‐mossy transition and to clarify that the observed improvements stem not only from Ag NP incorporation but also from the introduction of TREN, density functional theory (DFT) calculations were carried out to compare Li adsorption and migration behaviors on Ni, Ag and TREN‐coated Ag surfaces (Figure S14). As shown in Figure S15, the Li adsorption energy becomes increasingly negative from Ni (−1.59 eV) to Ag (−1.99 eV) and further to TREN‐coated Ag (−2.08 eV), indicating progressively enhanced lithiophilicity. The relatively weak Li adsorption on Ni suggests a limited ability to stabilize adsorbed Li at the initial deposition stage, whereas Ag provides a more favorable surface for Li adsorption. Upon introducing TREN, the adsorption energy becomes even more negative, implying that the N‐containing TREN layer offers additional energetically favorable sites for Li binding. Consistent with this trend, the calculated surface work functions shown in Figure S16 decreased from 4.98 eV for Ni to 4.30 eV for Ag and further to 4.04 eV for TREN‐coated Ag, indicating a progressively more favorable electronic environment for Li reduction in the order of Ni < Ag < TREN‐coated Ag. These results collectively suggest that Ag provides both stronger Li affinity and a more favorable electronic environment for Li reduction than Ni, while the introduction of TREN further amplifies these effects by strengthening Li binding and lowering the work function. Therefore, the TREN‐coated Ag surface is expected to provide the most favorable thermodynamic environment for Li nucleation, while the following NEB analysis further clarifies how post‐adsorption Li migration is regulated on each surface.

To further clarify the post‐adsorption Li behavior, NEB calculations were carried out to evaluate Li diffusion on Ni, Ag, and TREN‐coated Ag surfaces (Figure S17). The Ni surface exhibited a high Li diffusion barrier of 0.67 eV, indicating that Li migration after adsorption is kinetically hindered. In contrast, the Ag surface showed an almost negligible diffusion barrier of 0.01 eV, suggesting facile surface diffusion of adsorbed Li on the lithiophilic Ag surface despite its stronger adsorption compared with Ni. Notably, the TREN‐coated Ag surface exhibited a distinct potential energy well of −0.09 eV near the N‐containing TREN sites, indicating that TREN provides energetically favorable sites for locally stabilizing adsorbed Li. These results suggest that Ag promotes facile lateral Li redistribution, while the N sites of TREN guide and stabilize Li at uniformly distributed nucleation sites, thereby enabling spatially regulated Li nucleation and suppressing localized dendritic growth.

Given that regulated nucleation and migration can significantly influence electrolyte decomposition pathways and the chemistry of the interphase formed during cycling, we next analyzed the SEI to correlate the deposition behavior with interfacial stability. To investigate the chemical composition of the SEI formed on the electrodes, ex situ X‐ray photoelectron spectroscopy (XPS) analysis was conducted after 10 CV cycles (Figure 2g‐i). For the bare Ni textile, the F 1s, N 1s, and Li 1s spectra revealed strong signals from electrolyte decomposition products such as CF_x_, SO_2_F^−^, and LiN_x_O_y_. The high content of these species usually indicates that the anion (e.g., TFSI^−^) or electrolyte additives (e.g., LiNO_3_) are being continuously and severely decomposed by highly reactive Li, leading to the formation of unstable intermediates and thereby suggesting incomplete or nonuniform SEI formation [62, 63]. In sharp contrast, these species were substantially suppressed on the 3‐Ag‐Ni textile. In particular, the N‐containing decomposition species, such as LiN_x_O_y_, markedly diminished after cycling on the 3‐Ag‐Ni textile, whereas the Li_3_N‐related component became dominant in the N 1s spectrum. More importantly, the additionally analyzed N 1s spectra after 100 cycles showed that this Li_3_N‐dominant feature was well maintained on the 3‐Ag‐Ni textile, with a negligible LiN_x_O_y_ contribution (Figure S18).

To directly determine whether the TREN‐derived interfacial chemistry causes persistent active‐Li consumption, coulometric titration time analysis (CTTA) was performed using a TREN‐coated Ni textile without Ag NPs, thereby isolating the effect of TREN (Figure S19). The TREN‐coated Ni textile exhibited a shorter first open‐circuit voltage (OCV) duration (τ_OCV,1_) than the bare Ni textile, indicating an additional initial interfacial reaction. From the second titration onward, however, the TREN‐coated Ni textile consistently exhibited longer OCV durations (τ_OCV,2_), demonstrating slower subsequent Li consumption. Consistently, the time required for the accumulated charge consumed by side reactions to reach 300 µAh cm^−^^2^ was ∼80.8 h for the TREN‐coated Ni textile, compared with ∼78.8 h for the bare Ni textile. These results demonstrate that, although TREN participates in a limited initial Li‐consuming reaction, it does not accelerate cumulative Li consumption.

This interpretation is further supported by the persistent Li_3_N‐dominant N 1s feature observed after cycling, indicating that the initial Li–N interfacial reaction is self‐limiting and leads to the formation of a stable Li_3_N/LiF‐rich passivating interphase. The resulting interphase effectively suppresses subsequent Li‐consuming side reactions while facilitating reversible Li plating/stripping. Consequently, the lithiophilic interfacial modification of the host electrode regulates interfacial reaction pathways, suppresses excessive electrolyte‐anion decomposition, and mitigates parasitic active‐Li consumption.

### 2.3 Fabrication of a Lithiophilic Interfacial Interaction‐Mediated Separator

Based on the enhanced electrode stability provided by the lithiophilic [TREN/Ag NP]_3_ multilayer‐assembled Ni textile, we aimed to further improve the anode performance by modifying the pristine separator. Although the [TREN/Ag NP]_3_ multilayers deposited on the Ni textile‐based host electrode significantly enhance interfacial lithiophilicity and charge transfer efficiency at the electrode surface, the overall electrochemical performance remains constrained by sluggish Li^+^ ion transport through the separator and competitive anion migration.

To address this limitation, we engineered lithiophilic polymer layers consisting of COOH‐functionalized PAA and NH_2_‐functionalized PEI onto the surface of a glass fiber separator, which is mainly composed of SiO_2_. Generally, glass fiber separators possess substantially higher porosity (typically >85–90%) than polypropylene separators (∼40%), enabling greater electrolyte uptake and providing more abundant ion‐transport pathways. These characteristics help mitigate concentration polarization and local electrolyte depletion, thereby promoting homogeneous Li‐ion transport and suppressing Li dendrite growth, even under relatively high current densities. In addition, the excellent electrolyte wettability of glass fiber, arising from the hydrophilic silanol (Si–OH) groups on its surface, further facilitates uniform electrolyte distribution throughout the separator.

**FIGURE 3:**
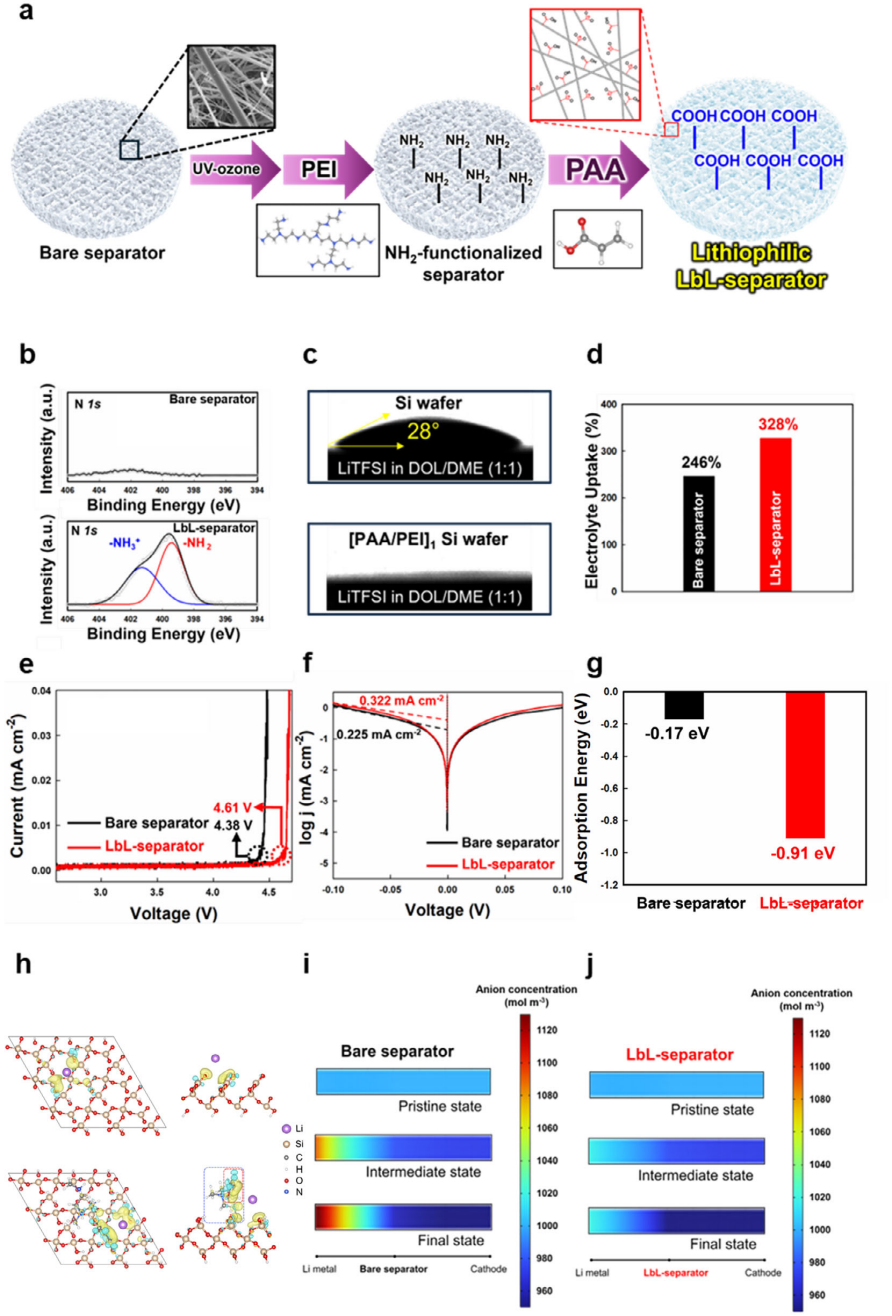
Fabrication of a Lithiophilic Interfacial Interaction‐Mediated Separator. (a) Schematic illustration of the preparation for lithiophilic interfacial interaction‐mediated separator. (b) Deconvoluted N 1s XPS spectra of the bare separator and LbL‐separator. (c) Electrolyte contact angle measurements of Si wafer and [PAA/PEI]_1_‐coated Si wafer. (d) Electrolyte uptake capabilities of bare separator and LbL‐separator. (e) Linear sweep voltammetry (LSV) profiles of bare separator and LbL‐separator. (f) Tafel plots of bare separator and LbL‐separator. (g) Comparison of Li^+^ adsorption energies for bare separator and LbL‐separator. (h) Charge density difference (CDD) analysis of Li ion adsorption on the bare SiO_2_ and PAA/PEI‐coated SiO_2_ surfaces, where red and blue dashed boxes highlight PAA and PEI, respectively. Each panel displays the top (left) and side views (right). Yellow and cyan regions indicate electron accumulation and depletion, respectively. One‐dimensional COMSOL‐simulated anion concentration profiles across the cell employing the (i) bare separator and (j) LbL‐separator at different stages of 2C discharge.

The presence of hydrogen‐bonding interactions between PAA and PEI, as well as between PEI and the glass‐fiber separator surface, facilitates the robust formation of a uniform [PAA/PEI]_1_ multilayer onto the separator surface (i.e., PAA/PEI/separator) (Figure 3a). In this case, the thickness of a single bilayer of PAA/PEI was measured to be approximately 2 nm, as determined from the assumption that the thickness of the PAA/PEI layer on the glass fiber separator is similar to that on SiO_2_/Si wafers, a hypothesis corroborated by ellipsometry measurements (Figure S20).

This unique design allows the [PAA/PEI]_1_‐separator with a PAA outermost layer—hereafter referred to as the ‘LbL‐separator’—to selectively enhance Li^+^ ion transfer while effectively suppressing anion transport. The successful formation of a single [PAA/PEI]_1_ multilayer on the separator surface was confirmed by XPS analysis (Figure 3b). The pristine separator exhibited no detectable N 1s signal, consistent with the absence of nitrogen‐containing functionalities. However, after [PAA/PEI]_1_ deposition, a distinct N 1s envelope emerged in the ∼399–402 eV region, confirming the incorporation of amine‐containing moieties. Notably, the N 1s spectrum could be deconvoluted into neutral amine (–NH_2_) and protonated/strongly interacting amine (–NH_3_^+^) components, with a markedly increased –NH_3_^+^ contribution compared to the PEI‐only reference (Figure S21). This redistribution toward the high‐binding‐energy component indicates strengthened interfacial interactions between PAA and PEI chains within the assembled multilayer, consistent with hydrogen bonding and/or acid–base ion‐pair formation. Notably, the PAA‐terminated layer‐by‐layer (LbL) chemistry is anticipated to be more pertinent for separator modification than a surface composed solely of amine‐functionalized PEI. The carboxyl group‐rich surface of PAA can facilitate charge‐selective ion transport by promoting Li^+^ ion migration while simultaneously restricting anion transport, thereby achieving a more uniform Li^+^ flux across the separator. Importantly, FE‐SEM images confirmed that the [PAA/PEI]_1_ coating preserved the porous fibrous morphology of the pristine separator without noticeable pore blocking (Figure S22), indicating that the ultrathin LbL layer can modify the separator surface while maintaining its intrinsic ion‐transport pathway.

The key advantage of this strategy lies in achieving enhanced electrochemical properties through a single, ultrathin coating of the lithiophilic [PAA/PEI]_1_ interlayer on the separator surface. However, both the bare glass fiber separator and [PAA/PEI]_1_ interlayer‐coated glass fiber separator exhibit near‐complete electrolyte wetting owing to their highly porous structure, regardless of the underlying difference in surface hydrophilicity. Therefore, electrolyte contact angle measurements were conducted on flat Si wafers using 1 M LiTFSI in a DOL/DME mixture, in order to investigate only the intrinsic electrolyte affinity of the [PAA/PEI]_1_ bilayer independent of structural geometry (Figure 3c). The [PAA/PEI]_1_‐coated Si wafer exhibited significantly improved electrolyte wettability compared to the bare Si wafer, reflecting the enhanced surface affinity induced by the abundant lithiophilic polar functional groups within the multilayer. This improved wettability provides model‐surface evidence that the [PAA/PEI]_1_ interlayer can contribute to favorable electrolyte affinity at the separator–electrolyte interface. Beyond interfacial wettability, the polar groups in the LbL‐separator facilitate capillary‐driven electrolyte infiltration into the separator pores, thereby enhancing electrolyte uptake and promoting uniform Li^+^ transport. Consequently, the electrolyte uptake, calculated using Equation (2), increased from 246% for the bare separator to 328% for the LbL‐separator (Figure 3d), where W*_o_* ​ and W*_t_* denote the separator's weight before and after electrolyte absorption, respectively.

(2) $$\mathrm{Electrolyte}\;\mathrm{uptake}\;=\frac{W_{t}-W_{o}}{W_{o}}\;\times \;100\%$$

This increase in uptake indicates superior electrolyte affinity, which is beneficial for lowering internal ionic resistance and ensuring prolonged cycling stability.

The electrochemical stability of the separators was further evaluated by linear sweep voltammetry (LSV). As shown in Figure 3e, the LbL‐separator exhibited a delayed onset of oxidative current at ∼4.61 V, compared to ∼4.38 V for the bare separator, indicating enhanced interfacial oxidative stability. This phenomenon is attributed to the complementary roles of PAA and PEI within the ultrathin interlayer. Specifically, strong Li^+^ coordination by the polar functional groups of the LbL‐separator reduces the local activity of oxidative anions (e.g., TFSI^−^) near the separator–electrolyte interface, while the hydrogen‐bonded PAA/PEI network stabilizes the interfacial structure and promotes homogeneous Li^+^ transport, thereby suppressing anion‐driven electrolyte decomposition at elevated voltages [64]. Collectively, these features suggest the feasibility of achieving high energy density through compatibility with high‐voltage cathode materials.

Additionally, Tafel analysis was conducted to evaluate the kinetic advantages of the LbL‐separator (Figure 3f). The exchange current density (j_0_) increased from 0.225 mA cm^−^^2^ for the pristine separator to 0.322 mA cm^−^^2^ for the LbL‐separator, indicating substantially improved charge‐transfer kinetics at the electrode–electrolyte interface. This enhanced interfacial behavior, combined with the improved oxidative stability evidenced by the LSV results, underscores the ability of the LbL‐separator to facilitate uniform Li deposition and support high‐performance, durable LMBs.

To verify whether these favorable separator properties translate into improved Li plating/stripping stability while decoupling the effect of electrode‐side lithiophilicity, Li | separator | Ni foil half‐cells were assembled by varying only the separator component. The EIS spectra showed that the PAA/PEI‐modified glass fiber separator exhibited a reduced charge transfer resistance (*R*_ct_) value compared with the bare glass fiber separator, indicating decreased interfacial resistance (Figure S23a). The PAA/PEI‐modified glass fiber separator exhibited more stable Li plating/stripping behavior than the bare glass fiber separator (Figure S23b), confirming that the ultrathin PAA/PEI interlayer contributes to stable Li metal interfacial reactions.

The chemical stability of the PAA/PEI interlayer during repeated Li plating/stripping was first examined by post‐cycling N 1s XPS analysis of the LbL‐separator after 100 cycles at 3 mA cm^−^^2^ and 1 mAh cm^−^^2^. The analysis revealed the persistent presence of both –NH_2_ and –NH_3_^+^ components (Figure S24), indicating that the PAA/PEI interlayer largely preserves its chemical integrity during repeated Li plating/stripping.

To further determine whether the polar functionalities within the LbL coating induce additional irreversible Li consumption, coulometric titration time analysis (CTTA) was performed using Li | separator | Ni textile cells while varying only the separator component (Figure S25). The cell employing the LbL‐coated separator consistently exhibited longer open‐circuit durations (τ_OCV,2_) than the cell with the bare separator from the first titration onward. Consistently, the time required for the cumulative side‐reaction charge (qΣ) to reach 300 µAh cm^−^^2^ increased from ∼78.8 h for the bare separator to ∼89.6 h for the LbL‐separator. These results demonstrate that the PAA/PEI interlayer does not increase irreversible Li consumption despite the presence of carboxyl and amine functionalities.

Taken together, the post‐cycling XPS and CTTA results demonstrate that the PAA/PEI LbL coating remains chemically stable during repeated Li plating/stripping and does not serve as a continuous Li‐consuming pathway. Instead, the polar interlayer suppresses parasitic Li‐consuming side reactions, thereby stabilizing the Li‐metal interface during prolonged cycling.

Furthermore, to examine the practical applicability of this separator‐side modification beyond glass fiber substrates, the same PAA/PEI assembly strategy was applied to a surface‐activated commercial PP separator. First, X‐ray photoelectron spectroscopy (XPS) analysis was performed to verify the successful assembly of the PAA/PEI multilayer on the PP separator (Figure S26). Consistent with the PAA/PEI‐modified glass fiber separator (i.e., the LbL separator), the PAA/PEI‐modified PP separator exhibited distinct contributions corresponding to protonated amine (‐NH_3_^+^) and neutral amine (NH_2_) species in the N 1s spectrum. The coexistence of these ‐NH_3_^+^ and NH_2_ components, originating from electrostatic interactions and hydrogen‐bonding interactions between the amine groups of PEI and the carboxyl groups of PAA, provides clear evidence for the successful formation of the PAA/PEI multilayer on the PP separator.

EIS analysis further revealed that the PAA/PEI‐modified PP separator showed a smaller *R*_ct_ value than the bare PP separator (Figure S27a), suggesting improved interfacial kinetics after PAA/PEI modification. The PAA/PEI‐modified PP separator also showed improved half‐cell stability compared with the bare PP separator in the same Li | separator | Ni foil configuration (Figure S27b), indicating that this separator‐side interfacial modification can be extended to commercially relevant polyolefin separator platforms.

To directly visualize how the separator‐side modification affects Li deposition morphology, ex situ FE‐SEM imaging was further conducted in Li | Ni foil half‐cells assembled with either the bare separator or the LbL‐separator at increasing plated capacities of 1, 3, and 5 mAh cm^−^^2^ (Figure S28). The Ni surface with the bare separator exhibited rough and non‐uniform Li deposits with pronounced protrusive features over the entire plating process, indicating localized Li nucleation and uncontrolled growth. In contrast, the LbL‐separator maintained a relatively smooth and compact deposition morphology even at higher plated capacities, suggesting that the modified separator enables a more homogeneous interfacial Li‐ion flux and effectively suppresses the development of irregular protrusions during Li plating.

In addition to the electrochemical evidence, we sought to clarify the molecular‐level origin of the improved interfacial kinetics enabled by the LbL‐separator. Specifically, density functional theory (DFT) calculations were performed to investigate how the PAA/PEI coating alters the Li^+^‐ion affinity and local electronic environment of the separator surface (Figure S29). As shown in Figure 3g, bare SiO_2_ exhibits a Li^+^ adsorption energy of −0.17 eV, indicating the weak intrinsic Li^+^ affinity of the bare surface, whereas PAA/PEI‐coated SiO_2_ yields a more negative value of −0.91 eV, demonstrating that the polymer coating renders Li^+^ adsorption energetically favorable. The charge density difference (CDD) analysis reveals pronounced electron accumulation around the PAA/PEI monomers in the vicinity of the Li^+^. This localized charge redistribution generates an electrostatic ion‐dipole interaction stronger than that on the bare SiO_2_ surface, leading to a highly stabilized adsorption energy and enhanced lithiophilicity of the PAA/PEI‐modified separator (Figure 3h).

To further clarify the macroscopic impact of the LbL‐separator on ionic transport dynamics under high‐rate conditions, 1D COMSOL Multiphysics simulations were conducted (Table S1) [65]. Figure 3i,j depict the evolution of the anion concentration profiles for cells with bare and LbL‐separators during a 2C discharge. The bare separator displays a steep concentration gradient, characterized by significant anion accumulation near the lithium metal side and depletion toward the cathode side as discharge progresses, indicative of severe concentration polarization due to insufficiently suppressed anion migration. In contrast, the LbL‐separator maintains a substantially more uniform anion distribution across the cell, demonstrating effective suppression of anion migration. This behavior is consistent with the markedly higher Li^+^ ion transference number of the LbL‐separator relative to that of the bare separator, which is attributed to the COO^−^‐functionalized outermost PAA layer that promotes selective Li^+^ ion conduction while inhibiting anion migration. Consequently, the LbL‐separator effectively alleviates concentration polarization and preserves a more stable ionic environment even at a high 2C rate. Combined with the improved wettability and electrochemical stability revealed by the preceding analyses, these results collectively demonstrate that the LbL‐separator regulates ion transport by suppressing anion migration and mitigating concentration polarization under high‐rate operation.

### 2.4 Symmetric Cell With Dual‐Interface Modification

**FIGURE 4:**
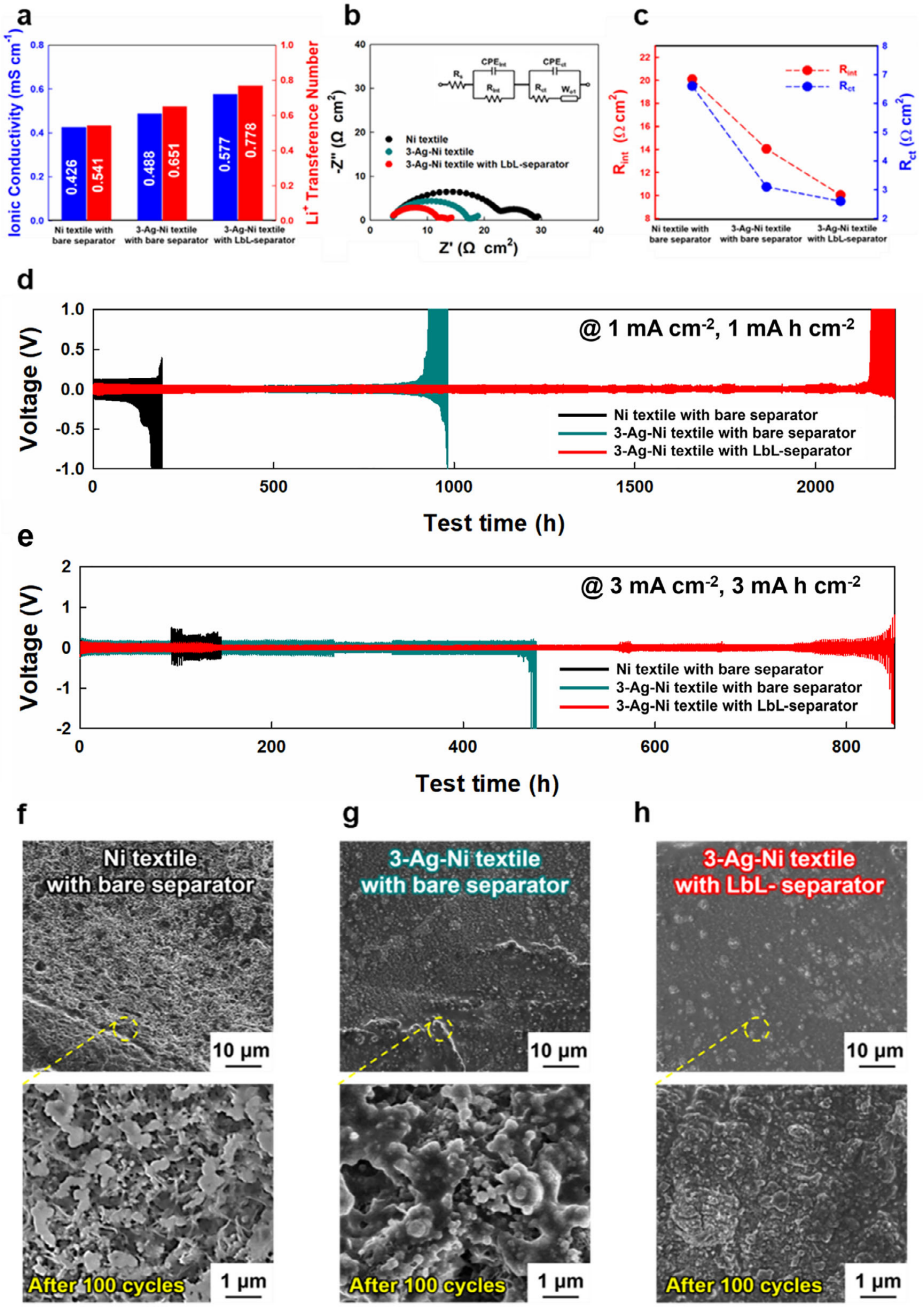
Symmetric Cell with Dual‐Interface Modification. (a) Comparison of ionic conductivity and Li^+^ transference numbers (*t*_Li+_) for Ni textile and 3‐Ag‐Ni textile anodes with and without the LbL‐separator. (b) Nyquist plots and the corresponding equivalent circuit for the bare Ni textile, 3‐Ag‐Ni textile, and dual‐modified symmetric cells. (c) Comparison of *R*_int_ and *R*_ct_ values of the symmetric cells with bare or modified components. Galvanostatic cycling profiles of the symmetric cells at (d) 1 mA cm^−2^, 1 mAh cm^−2^ and (e) 3 mA cm^−2^, 3 mAh cm^−2^. Planar FE‐SEM images of Li deposition morphologies after 100 plating/stripping cycles at 3 mA cm^−^^2^ and 1 mAh cm^−^^2^ for cells employing (f) bare Ni textile | bare separator, (g) 3‐Ag‐Ni textile | bare separator, and (h) dual‐modified 3‐Ag‐Ni textile | LbL‐separator cells after cycling.

To investigate the synergistic effect of the *n*‐Ag‐Ni textile anode and LbL‐separator on the electrochemical properties of LMB systems, a symmetric cell configuration composed of 3‐Ag‐Ni textile | LbL‐separator | 3‐Ag‐Ni textile was first employed to evaluate the Li^+^ transference number and overall charge transport efficiency. As shown in Figure 4a, the introduction of the LbL‐separator dramatically increased the Li^+^ transference number to 0.778—substantially higher than those of the Ni textile | bare separator | Ni textile configuration (0.541) and the 3‐Ag‐Ni textile | bare separator | 3‐Ag‐Ni textile (0.651) configurations (Figure S30). Notably, the 3‐Ag‐Ni textile | PEI‐coated separator | 3‐Ag‐Ni textile cell exhibited a Li^+^ transference number of 0.654 (Figure S31), which was nearly identical to that of the 3‐Ag‐Ni textile | bare separator | 3‐Ag‐Ni textile cell, whereas the LbL‐separator markedly increased the value to 0.778. These results demonstrate that the amine‐rich PEI coating alone does not significantly improve Li^+^‐selective transport, whereas the PAA‐containing LbL chemistry plays a critical role in suppressing anion migration and homogenizing Li^+^ flux. Additionally, the ionic conductivities of the cells exhibited a trend similar to that of the Li^+^ transference number. This concurrent enhancement of both parameters highlights the intrinsic advantage of the dual‐interface modification in promoting more uniform and efficient charge transport within the cell.

Beyond the promising bulk ion transport behavior, EIS measurements were further performed to evaluate the interfacial charge transfer kinetics of the dual‐interface modification systems, a key parameter governing Li nucleation at the electrode interface (Figure 4b,c). Notably, the 3‐Ag‐Ni textile | LbL‐separator | 3‐Ag‐Ni textile cells exhibited a remarkably low *R*_ct_ of 2.5 Ω cm^2^ and interfacial resistance (*R*_int_) of 10.1 Ω cm^2^, substantially lower than those of the *n*‐Ag‐Ni textile‐based symmetric cells assembled with a bare separator (Figure S32). These results clearly demonstrate that the synergistic combination of LbL‐assembled Ag NP multilayers on the Ni textile current collector and the PAA/PEI bilayers on the separator simultaneously enhances Li^+^ transport, reduces interfacial resistance, and promotes uniform Li nucleation.

Based on these observations, we evaluated the cycling stability of these symmetric cells under a constant current density of 1 mA cm^−^^2^ and a capacity of 1 mAh cm^−^^2^ (Figure 4d; Tables S2 and S3). Pronounced differences in overpotential evolution and cycling lifetime were observed. The bare Ni textile exhibited rapid voltage fluctuations and progressively increasing overpotentials, leading to early cell failure within 200 h. This poor stability is attributed to insufficient lithiophilicity, resulting in interfacial instability, uneven Li nucleation, and subsequent dendritic growth. In contrast, the 3‐Ag‐Ni‐based symmetric cell maintained stable cycling for over 900 h with suppressed overpotentials, yet the absence of separator modification eventually limited long‐term cycle retention, identifying the separator interface as a critical factor for further expanding cycle life. Impressively, the 3‐Ag‐Ni textile | LbL‐separator | 3‐Ag‐Ni textile cell with dual‐interface modification demonstrated exceptional long‐term stability, sustaining >2200 h of cycling without noticeable overpotential fluctuation or short‐circuiting. Notably, this stability persisted even under elevated conditions. Under 3 mA cm^−^^2^ and 3 mAh cm^−^^2^, the symmetric cell employing the 3‐Ag‐Ni textile | LbL‐separator | 3‐Ag‐Ni textile configuration delivered stable cycling for over 800 h without noticeable voltage polarization (Figure 4e). The superior stability of the LbL‐separator compared with the PEI‐coated separator further confirms that carboxylate‐assisted Li^+^ transport regulation is more effective than amine‐only surface modification for separator‐side stabilization (Figure S33). Even at a higher current density of 5 mA cm^−^^2^ and 1 mAh cm^−^^2^, the 3‐Ag‐Ni textile | LbL‐separator | 3‐Ag‐Ni textile cell maintained stable operation for over 600 h, again with negligible polarization growth (Figure S34). The cycling stability profiles of all symmetric cells are presented up to their final cell failure stage, while the localized enlarged views in Figure S35 verify that stable voltage polarization was preserved throughout the main cycling region before cell failure. These observations confirm that the cells maintained stable operation without premature short circuit. Overall, these results clearly demonstrate that dual‐interface modification is highly effective in enhancing the electrochemical robustness of LMA‐based battery applications.

To further elucidate how dual‐interface modification influences Li deposition morphology, ex situ FE‐SEM imaging was conducted after 100 plating/stripping cycles at 3 mA cm^−^^2^ and 1 mAh cm^−^^2^. As shown in Figure 4f, the bare Ni textile exhibited uncontrolled, dendritic Li deposits with sharp protrusions—morphologies indicative of poor lithiophilicity and highly heterogeneous nucleation. Such unstable deposits accelerate cell degradation and increase the risk of internal short circuits, underscoring the inherent limitations of the unmodified Ni textile. In stark contrast, the 3‐Ag‐Ni textile facilitated smooth and densely packed Li deposition (Figure 4g), confirming the beneficial effect of Ag‐induced lithiophilicity. Most notably, the combined use of the 3‐Ag‐Ni textile and the LbL‐separator resulted in a remarkably uniform, dense, mossy Li morphology even after 100 cycles (Figure 4h), validating the strong synergistic advantage of dual‐interface engineering.

### 2.5 Asymmetric Cells With Dual‐Interface Modification

**FIGURE 5:**
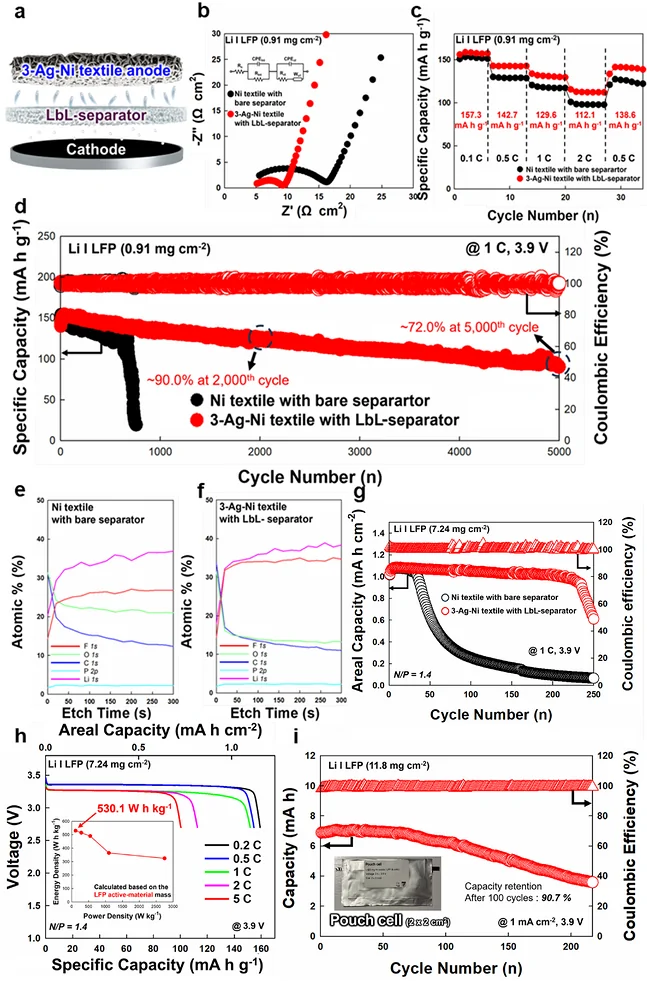
Asymmetric Cells with Dual‐Interface Modification. (a) Schematic illustration for the preparation of asymmetric full cell composed of 3‐Ag‐Ni textile anode and LbL‐separator. (b) Nyquist plots and the corresponding equivalent circuit (inset) for LFP‐based full cells with bare and dual‐interface modified components. (c) Rate capability tests of the full cells at 0.1 C to 2 C. (d) Galvanostatic cycling performance of the bare Ni textile and dual‐modified cells at 1 C (cathode loading: 0.91 mg cm^−2^) with an upper cutoff voltage of 3.9 V. XPS depth profiles of the SEI layer formed on (e) the bare Ni textile and (f) the dual‐modified 3‐Ag‐Ni textile after the 100^th^ GCD cycle. (g) Long‐term cycling stability under lean‐Li conditions (N/P ratio of 1.4). (h) Rate‐dependent voltage profiles of the 3‐Ag‐Ni textile | LbL‐separator | LFP full cell under high‐loading/low‐N/P conditions. The inset shows the corresponding Ragone plot comparing energy and power densities calculated based on the mass of LFP active material under a high LFP loading of 7.24 mg cm^−^^2^. (i) Cycling performance and Coulombic efficiency of the 3‐Ag‐Ni textile anode | LbL‐separator | LFP cathode pouch cell with a cathode active‐material loading of 11.8 mg cm^−^^2^, tested at 1 mA cm^−^^2^ within a voltage range of 2.5–3.9 V. The inset shows a digital image of the assembled pouch cell.

To validate the practical applicability of the proposed dual‐interface modification strategy, asymmetric full cells were assembled by pairing the 3‐Ag‐Ni textile anode and LbL‐separator with an LFP or NCM cathode (i.e., the 3‐Ag‐Ni textile | LbL‐separator | cathode) (Figure 5a). The interfacial properties of the full cells were first evaluated by EIS measurement (Figure 5b; Figure S36). The full cell employing the 3‐Ag‐Ni textile with the LbL‐separator showed a markedly smaller semicircle in the Nyquist plot than the cell using the bare Ni textile with the bare separator, corresponding to a pronounced reduction in the overall interfacial resistance from 11.12 to 4.21 Ω cm^2^. In addition, the modified cell exhibited a lower *R*_s_, further supporting improved ion transport characteristics. These results indicate that the dual‐interface modification of the electrode and separator effectively alleviates interfacial polarization and facilitates Li^+^ transport under practical full cell conditions.

These improved interfacial kinetics resulted in superior rate capability. As shown in Figure 5c, the dual‐modified full cell delivered higher specific capacities than the bare Ni textile‐employed cell across all tested C‐rates, from 0.1 C to 2 C, demonstrating the effectiveness of the dual‐interface modification in enabling stable operation under fast charge/discharge conditions. To further assess the chemical stability of the full‐cell interface under non‐operating conditions, the self‐discharge behavior of LFP‐based full cells was monitored during a 24 h open‐circuit rest period after charging to 3.9 V (Figure S37). The bare Ni textile cell with the unmodified bare separator exhibited a more significant voltage decay, reflecting continuous parasitic reactions and unstable interfacial chemistry during the rest period. In contrast, the 3‐Ag‐Ni textile | LbL‐separator | LFP full cell maintained a more stable voltage profile with reduced self‐discharge, indicating that the dual‐interface modification effectively mitigates spontaneous electrolyte/Li consumption and preserves the charged state of the cell. Owing to these improved interfacial kinetics and suppressed self‐discharge behavior, the 3‐Ag‐Ni textile | LbL‐separator | LFP full cell with an LFP loading of 0.91 mg cm^−2^ retained 72% of its initial capacity after 5000 cycles at 1C, while maintaining a Coulombic efficiency close to 100%. In stark contrast, the cell employing the bare Ni textile with a bare separator experienced rapid degradation and failed prematurely (Figure 5d; Figure S38). It is worth noting that our dual‐interface modification strategy could effectively be applied to typical layered transition‐metal oxide cathodes, such as LiNi_0_._8_Co_0_._1_Mn_0_._1_O_2_ (NCM), resulting in significantly improved electrochemical performance without compromising the intrinsic cathode behavior (Figure S39).

To further elucidate the origin of the improved full‐cell durability, XPS depth profiling was performed on the cycled anodes retrieved from LFP‐based asymmetric full cells after 100 charge–discharge cycles. Notably, the Ni textile cell using a bare separator displayed an increasing O‐rich signature with etching depth, implying the severe formation and accumulation of organic/oxygenated decomposition species. Concurrently, it exhibited a relatively lower fluorine (F) content that failed to remain pronounced in the deeper regions (Figure 5e). In contrast, the dual‐modified cell (3‐Ag‐Ni textile | LbL‐separator) displayed opposite trends for the O and F elements. It exhibited a relatively suppressed O‐rich signature with increasing depth, indicating reduced formation of such decomposition species. Furthermore, it maintained a higher F content throughout the etching depth, with the F fraction increasing and remaining significantly more pronounced in deeper regions (Figure 5f). Importantly, high‐resolution F 1s analysis further revealed that this F enrichment primarily originated from LiF formation rather than the accumulation of LiPF_6_‐derived fluorinated species (Figure S40). Compared with the bare‐separator‐based Ni textile cell, the dual‐modified cell exhibited a markedly stronger LiF contribution and a reduced high‐binding‐energy F component assigned to Li_x_PO_y_F_z_/P–F species. These results indicate that the dual‐interface modification not only suppresses oxygenated organic decomposition but also redirects interphase chemistry toward a robust LiF‐rich SEI. The formation of this LiF‐dominant interphase is expected to enhance mechanical integrity, suppress parasitic electrolyte decomposition, and stabilize Li plating/stripping, thereby accounting for the reduced polarization and improved cycling stability observed in Figure 5d.

To further substantiate these findings, long‐term cycling was performed under a more demanding condition with a high‐LFP loading of 7.24 mg cm^−^^2^ and a low N/P ratio of 1.4 (Figure 5g). Notably, the 3‐Ag‐Ni textile | LbL‐separator | LFP full cell exhibited excellent cycling stability, retaining ∼92.0% of its initial capacity even after 235 GCD cycles at 1C, in stark contrast to the bare Ni textile‐based cell, which showed rapid capacity fading before 40 cycles. To further evaluate the cell performance under lean‐electrolyte conditions, the electrolyte volume was reduced from 100 to 50 µL (Figure S41). The cells were assembled with the same LFP loading and N/P ratio as those in Figure 5g. Notably, the dual‐modified cell still exhibited improved cycling stability under the limited‐electrolyte condition (E/C ratio of 23 µL/mAh), confirming that the enhanced performance does not simply result from excess electrolyte addition but rather originates from the dual‐interface modification. This stable cycling behavior further confirms the superior reversibility of the dual‐modified cell architecture, which effectively utilizes the Li source to form a high‐quality SEI while minimizing parasitic consumption. Post‐cycling FE‐SEM analysis further supported this interpretation (Figure S42). After cycling, the Ni textile | bare separator‐based LFP full cell exhibited heavily accumulated, irregular dendritic Li deposits, whereas the 3‐Ag‐Ni textile | LbL‐separator‐based LFP full cell maintained a comparatively uniform and conformal Li‐covered fibrous morphology even after prolonged cycling, indicating more regulated Li deposition and improved interfacial stability.

To further evaluate the structural reversibility of Li plating/stripping, ex situ XRD analysis was performed on the 3‐Ag‐Ni textile anodes paired with the LbL separator at three representative electrochemical states: after the first lithiation, after the first delithiation, and after prolonged cycling to end‐of‐life (Figure S43). After the first lithiation, the Li(110) reflection was clearly observed together with a LiF peak, confirming deposition of metallic Li and the initial formation of an inorganic SEI. Following the first delithiation, no discernible crystalline Li reflections were detected, whereas the LiF peak remained, indicating efficient stripping of the deposited Li while preserving the inorganic SEI. Even after prolonged cycling to end‐of‐life state, no detectable reflections corresponding to crystalline metallic Li were observed, with only weak signals arising from inorganic SEI species, including LiF, Li_2_O, and LiOH. These results provide direct structural evidence that the dual‐interface architecture enables highly reversible Li plating/stripping while suppressing the accumulation of crystalline inactive Li detectable by XRD during extended cycling. This interpretation is further supported by previous XRD studies demonstrating that residual Li(110) reflections after discharge signify incomplete Li stripping and dead‐Li accumulation, whereas Li(110)‐oriented deposition on 3D hosts promotes more homogeneous and reversible Li plating/stripping [66, 67].

To further elucidate the through‐thickness degradation behavior, post‐mortem cross‐sectional FE‐SEM analysis was conducted on textile anodes harvested from full cells cycled to their respective end‐of‐life under different LFP loading conditions (Figure S44a). The bare Ni textile paired with the bare separator exhibited dense, spatially heterogeneous deposits that extensively filled the internal porous framework, indicative of nonuniform Li plating/stripping accompanied by the progressive accumulation of inactive Li and electrolyte‐decomposition products. In contrast, the 3‐Ag‐Ni textile paired with the LbL separator maintained a substantially more homogeneous deposit distribution throughout the fibrous framework, with significantly less localized accumulation and pore blockage, even at the high LFP loading of 7.24 mg cm^−^^2^.

Cross‐sectional EDX elemental mapping of the cycled 3‐Ag‐Ni textile from the high‐loading full cell further revealed continuous Ni and Ag signals across the entire electrode thickness, indicating that the Ag‐containing interfacial layer remained uniformly distributed without discernible macroscopic redistribution during cycling (Figure S44b). These post‐mortem analyses collectively demonstrate that the dual‐interface architecture effectively suppresses heterogeneous deposit accumulation while preserving the internal porous structure of the textile anode, thereby contributing to stable Li deposition under practically relevant high‐loading conditions.

As a result, the 3‐Ag‐Ni textile | LbL‐separator‐based full cells delivered maximum energy and power densities of 530.1 Wh kg^−^^1^ and 2748.8 W kg^−^^1^, respectively, calculated based on the mass of LFP active material under a high LFP loading of 7.24 mg cm^−^^2^ (Figure 5h). In addition, at a N/P ratio of 1.4, the specific energy calculated based on the combined mass of the LFP active material and pre‐deposited Li inventory reached 499 Wh kg^−1^, surpassing those of previously reported LMBs (Table S4).

Furthermore, we calculated the energy density of the electrode using the total mass including LFP, conductive additive, binder, and current collector. In this case, the mass contributions were 12.8 mg of LFP active material, 3.2 mg of conductive additive and binder, 9.0 mg of Al current collector, 0.79 mg of Li inventory for N/P = 1.4, 60.0 mg of 3‐Ag‐Ni textile host, 40.0 mg of glass‐fiber separator, and 100.0 mg of electrolyte. Based on a total component mass of approximately 225.8 mg, the corresponding component‐level specific energy was estimated to be approximately 30.1 Wh kg^−^^1^ under the present high‐loading coin‐cell configuration.

Collectively, these full cell results clearly demonstrate that the synergistic combination of a lithiophilic 3D electrode and a functional separator enables stable Li metal behavior, establishing it as a key strategy for realizing high‐performance, long‐lasting LMBs.

To extend the applicability beyond coin‐cell configurations, a pouch cell was fabricated using the 3‐Ag‐Ni textile anode and the LbL‐separator, underscoring the scalability and practical potential of dual‐interface modification. When paired with a LFP cathode featuring an active material loading of 11.8 mg cm^−^^2^, the pouch cell exhibited good cycling stability, retaining approximately 90.7% of its initial capacity after 100 cycles (Figure 5i). The shorter cycle life observed under higher cathode loading and lower N/P ratio can be attributed to the combined effects of a limited Li reservoir and increasingly demanding cell‐level conditions. Specifically, the higher cathode loading requires more extensive Li plating/stripping during each cycle, while the reduced N/P ratio limits the available Li inventory to compensate for irreversible Li loss. These harsher conditions are expected to accelerate the accumulation of electrically isolated (dead) Li and the associated depletion of active Li inventory, thereby promoting continuous side reactions at the Li/electrolyte interface, increased cell polarization, and ultimately premature cell failure. Nevertheless, the dual‐interface‐modified cells consistently outperformed their bare counterparts, underscoring the robustness of the lithiophilic interface design across a range of practically relevant operating conditions. This demonstration in a practical cell format confirms that the dual‐interface modification strategy—combining an ultrathin, lithiophilic TREN/Ag NP multilayer with a functional PAA/PEI‐assembled separator—provides a highly effective pathway for developing dendrite‐free, high‐performance Li metal anodes. Therefore, we believe that this approach holds strong promise for enabling the next generation of advanced LMBs.

## 3 Conclusions

In this study, we report high‐performance LMBs enabled by a dual lithiophilic interfacial interaction–mediated assembly strategy. This rationally designed approach offers a robust and effective solution to suppress uncontrolled Li dendrite growth during prolonged plating/stripping processes. Specifically, ultrathin, nanoporous, lithiophilic [TREN/Ag NP]_3_ multilayers were integrated onto a 3D porous Ni textile, while a conformal [PAA/PEI]_1_ bilayer was deposited onto the separator, collectively constructing a synergistically engineered electrode–separator architecture. The optimized [TREN/Ag NP]_3_ multilayers and [PAA/PEI]_1_ bilayer exploit ligand‐exchange–induced covalent interactions and strong hydrogen bonding, respectively, thereby markedly enhancing charge transport, homogenizing current distribution across both interfaces, and preserving exceptional structural integrity during cycling.

A key advantage of this approach lies in the ability to introduce ultrathin functional coatings—collectively below 50 nm across both interfaces—without incorporating electrochemically inactive components. The resulting uniformly lithiophilic interfaces promote stable Li nucleation within the available pore structure, minimize internal resistance, and substantially improve energy efficiency, even under high‐rate operation. Importantly, this nanoscale chemical and physical control enables precise regulation of Li^+^ flux and interfacial stability, thereby overcoming fundamental limitations associated with conventional LMA designs.

As a result, the optimized symmetric cell (3‐Ag‐Ni textile | LbL‐separator | 3‐Ag‐Ni textile) achieved exceptional cycling durability of 2200 h at 1 mA cm^−^^2^ and 1 mAh cm^−^^2^, with a remarkably low overpotential of 33 mV—far surpassing the performance of symmetric cells employing traditional LMA architectures. Furthermore, the full cells paired with LFP cathodes (i.e., 3‐Ag‐Ni textile | LbL‐separator | LFP) at an LFP loading of 0.91 mg cm^−2^ retained 72.0% of their initial capacity after 5000 cycles. When evaluated under more practically relevant conditions involving a higher LFP loading of 7.24 mg cm^−2^ and a limited Li inventory with an N/P ratio of 1.4, the full cells delivered a high LFP‐normalized specific energy of 530.1 Wh kg^−^^1^, calculated based on the mass of LFP active material. Notably, the resulting pouch cell delivered an initial capacity of 6.9 mAh and retained approximately 90.7% of its initial areal capacity of 1.7 mAh cm^−^^2^ over 100 cycles, demonstrating the feasibility of scaling this interfacial design to practical battery formats. Overall, the dual‐interface modification strategy presented herein—combining lithiophilic LbL‐assembled nanostructures with a mechanically compliant 3D host—offers precise control over Li deposition behavior and effectively mitigates dendrite formation. This work establishes a versatile and scalable framework for advancing next‐generation LMBs with high specific energy and long‐term operational stability.

## 4 Experimental Section/Methods

Detailed experimental procedures are provided in the Supporting Information.

## Author Contributions

C.L., D.N., Y.K., and J.C. conceived and designed the study. C.L. and D.N. performed the experiments and analyzed the experimental data. S.H.B. and J.K. performed the DFT calculations and analyzed the computational data under the supervision of S.B. B.K. also contributed to the electrochemical analyses and revision process. Y.S. assisted with the preparation and optimization of LFP cathodes. D.K., J.H.M., Y.J.C., and H.J.K. provided scientific input and contributed to data interpretation. Y.K. and J.C. supervised the overall project. C.L., D.N., Y.K., and J.C. wrote the manuscript. All authors discussed the results and commented on the manuscript.

## Conflicts of Interest

The authors declare no conflicts of interest.

## Supporting information




**Supporting File**: advs77494‐sup‐0001‐SuppMat.docx.

## Data Availability

The data that support the findings of this study are available on request from the corresponding author. The data are not publicly available due to privacy or ethical restrictions.
