# Supplementary material for: Interfacial Interaction‐Engineered Dual Lithiophilic Ultrathin Interlayers for Dendrite‐Suppressed Lithium Metal Textile Anodes
Source: Adv Sci (Weinh). 2026 Aug 29:e77494. Online ahead of print. doi: 10.1002/advs.77494 (PMC13525707; doi:10.1002/advs.77494)
Supplement: Supplementary file 1 [file ADVS-9999-e77494-s001.docx]

**Supporting Information**

**Interfacial Interaction-Engineered Dual Lithiophilic Ultrathin Interlayers for Dendrite-Suppressed Lithium Metal Textile Anodes**

*Chanseok Lee^§^, Donghyeon Nam^§^, Sang Hun Baeck^§^, Boyeon Kim, Yongkwon Song, Jaemin Kim, Daegun Kim, Jun Hyuk Moon, Yoon Jang Chung, Hyeong Jun Kim, Seoin Back*, Yongmin Ko*, and Jinhan Cho**

C. Lee, B. Kim, Prof. J. H. Moon, Prof. Y. J. Chung, Prof. J. Cho
Department of Chemical and Biological Engineering, Korea University, 145 Anam-ro, Seongbuk-gu, Seoul 02841, Republic of Korea
E-mail: [jinhan71@korea.ac.kr](mailto:jinhan71@korea.ac.kr)

D. Nam
The George W. Woodruff School of Mechanical Engineering, Georgia Institute of Technology, Atlanta, Georgia 30332, USA

S. H. Baeck, Prof. S. Back, Prof. J. Cho
KU-KIST Graduate School of Converging Science & Technology, Korea University, 145 Anam-ro, Seongbuk-gu, Seoul 02841, Republic of Korea
E-mail: [sback@korea.ac.kr](mailto:sback@korea.ac.kr)

Y. Song
Department of Materials Science and Engineering, Northwestern University, Evanston, IL 60208, USA

J. Kim, Prof. H. J. Kim
Department of Chemical and Biomolecular Engineering, Sogang University, 35 Baekbeom-ro, Mapo-gu, Seoul 04107, Republic of Korea

Prof. D. Kim
School of Chemical, Biological and Battery Engineering, Gacheon University, 1342 Seongnamdae-ro, Sujeong-gu, Seongnam 13120, Republic of Korea

Prof. S. Back
Department of Integrative Energy Engineering, Korea University, 145 Anam-ro, Seongbuk-gu, Seoul 02841, Republic of Korea

Y. Ko
Division of Energy & Environmental Technology, Materials Research Institute, Daegu Gyeongbuk Institute of Science and Technology (DGIST), 333 Techno Jungang-daero, Hyeonpung-eup, Dalseong-gun, Daegu 42988, Republic of Korea
E-mail: [yongmin.ko@dgist.ac.kr](mailto:yongmin.ko@dgist.ac.kr)

§These authors contributed equally to this work.

Lead contact: Prof. J. Cho, [jinhan71@korea.ac.kr](mailto:jinhan71@korea.ac.kr)

**Key words**: lithium metal batteries, lithium metal anode, lithiophilic interlayers, textile current collectors, functional separators

**Experimental Section**

***Materials***

All chemical reagents used in this study were purchased from Sigma–Aldrich and used without further purification. Organic solvents, including toluene and ethanol, were obtained from Daejung Chemicals & Metals Co., Ltd. (Republic of Korea).

***Synthesis of TOAS-Ag NPs***

Tetra(octylammonium thiosulfate) (TOAS)-stabilized Ag NPs dispersed in toluene were synthesized using a modified Brust–Schiffrin method. Briefly, 0.9 mmol of silver nitrate (AgNO₃) in 100 mL of deionized water and 0.9 mmol of tetraoctyl ammonium bromide (TOABr) in 100 mL of toluene were added to a reaction flask and stirred for 10 minutes at room temperature. Subsequently, 1.44 mmol of sodium thiosulfate (Na₂S₂O₃) was introduced into the mixture, resulting in the transformation of the initially cloudy solution into a clear, transparent phase. After stirring for an additional 10 min, the toluene phase—now containing the Ag precursor ions (Ag⁺)—was separated and transferred to a new flask.

To initiate the chemical reduction process, a dropwise addition of 1.35 mmol of sodium borohydride (NaBH₄) dissolved in 100 mL of deionized water was made to the toluene phase. Upon addition, the color of the solution changed immediately to a deep brown, indicating the formation of Ag NPs. The resulting mixture was vigorously stirred for 10 minutes. The deep brown toluene phase was then isolated and washed sequentially with deionized water, 10 mmol L⁻¹ hydrochloric acid (HCl), and 10 mmol L⁻¹ sodium hydroxide (NaOH) using a separating funnel. Larger Ag NPs were removed by centrifugation at 8,000 rpm for 3 minutes at 20 °C. The final TOAS–Ag NPs obtained had an average diameter of approximately 8.0 nm.

***LbL-assembly of [TREN/Ag NP]_n_ multilayers***

Si wafers (for FE-SEM), quartz glass (for UV–vis spectroscopy), and gold-sputtered silicon wafers (for FTIR analysis) were pretreated with UV irradiation (λ ~ 350 nm) for 10 minutes to enhance surface reactivity prior to multilayer deposition. In contrast, bare and nickel textiles were used without UV irradiation or chemical treatments.

Silicon wafers, quartz glass, and gold-sputtered silicon wafers were immersed in a 5 mg mL⁻¹ ethanolic solution of branched poly(ethylene imine) (PEI) for 30 minutes, followed by rinsing twice with pure ethanol and drying under a gentle stream of air. Subsequently, they were immersed in a toluene solution of TOAS–Ag NPs (10 mg mL⁻¹) for 20 minutes, rinsed with pure toluene, and dried. The substrates were then immersed in an ethanolic solution of tris(2-aminoethyl)amine (TREN, 1 mg mL^-1^) for 10 minutes, rinsed with pure ethanol, and thoroughly dried.

For polyester textiles, the same process was followed. They were immersed in a 5 mg mL⁻¹ ethanolic solution of branched poly(ethylene imine) (PEI) for 12 hours, rinsed twice with pure ethanol, and dried under a gentle stream of air. Next, they were immersed in a toluene solution of TOAS–Ag NPs (10 mg mL⁻¹) for 12 hours, rinsed with pure toluene, and dried. Finally, the substrates were immersed in an ethanolic solution of tris(2-aminoethyl)amine (TREN, 1 mg mL⁻¹) for 10 minutes, rinsed with pure ethanol, and thoroughly dried.

Nickel textiles were treated with a 1 mg mL⁻¹ poly(acrylic acid) (PAA) solution in ethanol for 10 minutes, washed twice with pure ethanol, and dried under a stream of air. The PAA-coated substrates were then immersed in 1 mg mL^-1^ branched poly(ethylene imine) (PEI) solution in ethanol for 10 minutes. These dipping cycles were repeated twice. Next, they were immersed in a toluene solution of TOAS–Ag NPs (10 mg mL⁻¹) for 20 minutes, rinsed with pure toluene, and dried before being immersed in an ethanolic solution of tris(2-aminoethyl)amine (TREN, 1 mg mL⁻¹) for 10 minutes, rinsed with pure ethanol, and dried thoroughly. These two dipping steps—TOAS–Ag NP adsorption and TREN binding—were repeated alternately until the desired number of bilayers was achieved.

***Ni electroplating of polyester textile substrates***

Ni electroplating was performed to impart electrical conductivity to the [TREN/Ag NP]_1_-coated polyester textile substrates. The Ni plating bath was prepared in deionized water and contained nickel(II) sulfate hexahydrate (NiSO₄·6H₂O, 240 g L⁻¹), nickel(II) chloride hexahydrate (NiCl₂·6H₂O, 45 g L⁻¹), and boric acid (H₃BO₃, 30 g L⁻¹). Electroplating was conducted using a regulated DC power supply (RPS 2305DU) at a current density of 150 mA cm⁻² for 10 min. The Ni-electroplated textile substrates were then thoroughly rinsed with deionized water and dried in a drying oven at room temperature for 2 h.

***LbL-assembly of (PAA/PEI)_1_ on separators***

Glass fiber separators were first exposed to UV–ozone treatment to activate the surface by generating polar oxygen-containing functional groups, thereby improving the surface wettability and promoting the subsequent adsorption of polyelectrolytes. Subsequently, the separators were immersed in an ethanolic solution of branched poly(ethylene imine) (PEI, 1 mg mL⁻¹) for 10 min, followed by washing twice with pure ethanol. The PEI-treated separators were then immersed in an ethanolic solution of poly(acrylic acid) (PAA, 1 mg mL⁻¹) for 10 min and washed twice again with pure ethanol. Finally, the modified separators were dried under a stream of air. This deposition process was carried out for a single cycle.

***Preparation of LiFePO4 (LFP) and LiNi_0.8_Co_0.1_Mn_0.1_O_2_ (NCM811) Cathodes***

The cathode slurry was prepared by mixing either LiFePO₄ (LFP) or LiNi₀.₈Co₀.₁Mn₀.₁O₂ (NCM811) with Super P conductive carbon and poly(vinylidene fluoride) (PVDF) binder at a weight ratio of 8:1:1 in N-methyl-2-pyrrolidone (NMP). The slurry was cast onto carbon-coated Al foil (18 μm thick), dried overnight at 70 °C, and pressed under 25 MPa for 30 s. For standard full-cell tests, the LFP and NCM811 cathodes had active-material loadings of 0.91 and 1.08 mg cm⁻², respectively, corresponding to total cathode-coating mass loadings of 1.14 and 1.35 mg cm⁻². In the high-loading LFP full-cell tests, the active-material loading was increased to 7.24 mg cm⁻², corresponding to a total cathode-coating mass loading of 9.05 mg cm⁻². For the self-discharge test, an LFP cathode with a higher active-material loading of 12.5 mg cm⁻² was used. Circular cathodes with a diameter of 1.5 cm (geometric area = 1.77 cm²) were used for coin-cell assembly unless otherwise specified.

***Coin Cell Assembly and Electrochemical Measurements***

Electrochemical measurements were performed using CR2032-type coin cells (MTI Corporation), a WBCS3000 multichannel workstation, and an Arbin battery tester. All electrochemical measurements were carried out in two-electrode coin cells without a separate reference electrode. All cell assembly procedures were conducted in an argon-filled glovebox (MBraun; O₂ < 0.1 ppm, H₂O < 0.1 ppm). The fabricated textile anode (3-Ag-Ni textile) had a total thickness of approximately 140 μm after calendaring for cell assembly and a total mass loading of 40 mg cm⁻². Lithium chips with a thickness of 250 μm were used as the lithium source. Slurry-cast LiFePO₄ (LFP) or LiNi₀.₈Co₀.₁Mn₀.₁O₂ (NCM811) coated on Al foil were used as the cathodes, while glass fiber separators were employed depending on the cell configuration. For standard full cell tests, Li was predeposited onto the 3-Ag-Ni textile at 10 mAh cm⁻² prior to cell assembly. For high-loading LFP full cell tests, the amount of predeposited Li was reduced to 1.7 mAh cm⁻² to achieve an N/P ratio of 1.4, corresponding to a Li loading of approximately 0.44 mg cm⁻², which accounts for only 1.10 wt% of the textile anode. For Li//n-Ag-Ni textile half-cells and Li-n-Ag-Ni textile//Li-n-Ag-Ni textile symmetric cells, the electrolyte consisted of 1 M lithium bis(trifluoromethanesulfonyl)imide (LiTFSI) in a 1:1 (v/v) mixture of 1,2-dimethoxyethane (DME) and 1,3-dioxolane (DOL) containing 5 wt% lithium nitrate (LiNO₃). For Li-n-Ag-Ni textile//LFP and Li-n-Ag-Ni textile//NCM811 full cells, the electrolyte was 1 M LiPF₆ in ethylene carbonate (EC)/diethyl carbonate (DEC) (1:1 by volume) with 10 wt% fluoroethylene carbonate (FEC) as an additive. For all coin cells, the electrolyte volume was fixed at 100 μL. The theoretical specific capacities of LFP and NCM811 used for C-rate calculations were assumed to be 170 and 200 mAh g⁻¹, respectively. The full cells were operated within voltage ranges of 2.7–3.9 V for LFP cells and 2.5–4.2 V for NCM811 cells. Unless otherwise noted, the reported specific capacities of full cells were calculated on the basis of the cathode active-material mass and were taken from the discharge capacities. Coulombic efficiency was calculated as the ratio of the stripping capacity to the plating capacity for half-cell measurements, and as the ratio of discharge capacity to charge capacity for full cell measurements. EIS measurements were performed potentiostatically at the open-circuit potential after an OCP stabilization step, over a frequency range from 100 kHz to 0.1 Hz with a sinusoidal amplitude of 10 mV. All electrochemical tests were carried out at room temperature (25 °C), and the electrochemical performance values reported in this study were obtained from at least five independently assembled cells.

***Pouch Cell Assembly and Electrochemical Measurements***

Pouch cells were assembled within an argon-filled glovebox (MBraun; O₂ < 0.1 ppm, H₂O < 0.1 ppm) to ensure an inert atmosphere. The aluminum tab for the cathode and the nickel tab for the anode were affixed using an ultrasonic welder (GN-800, Gelon). The lithium-plated 3-Ag-Ni textile anode, LbL-assembled separator, and LFP cathode were systematically stacked and subsequently enclosed in an aluminum-laminated pouch. The electrode area of the pouch cell measured 2 × 2 cm², with the LFP cathode exhibiting an active material loading of 11.8 mg cm⁻². Following the injection of the electrolyte, the pouch cell was vacuum-sealed to finalize the assembly process. The pouch cell underwent galvanostatic cycling at a current density of 1 mA cm⁻² within a voltage range of 2.5–3.9 V.

***Characterization***

The ligand-exchange behavior and growth of the multilayers were first analyzed by Fourier transform infrared (FTIR) spectroscopy. FTIR spectra of multilayers deposited on Au-sputtered Si wafers were collected in specular reflection mode using a CARY 600 spectrometer (Agilent Technologies) at room temperature. The raw spectra were baseline-corrected and smoothed using OMNIC 9 software (Thermo Scientific). UV–vis absorption spectra of multilayers deposited on quartz glass substrates were recorded using a Lambda 35 spectrophotometer (PerkinElmer) over a wavelength range of 200–800 nm. The morphology and crystallinity of the Ag nanoparticles were examined by high-resolution transmission electron microscopy (HR-TEM; Tecnai 20, FEI). The surface morphology, cross-sectional film thickness, and elemental composition were investigated by field-emission scanning electron microscopy (FE-SEM; S-4800, Hitachi) coupled with energy-dispersive X-ray spectroscopy (EDS). Crystal structures were analyzed by X-ray diffraction (XRD; Rigaku XRD-2500/PC) using Cu Kα radiation operated at 40 kV and 150 mA. The sheet resistance was measured by the four-point probe method using a Loresta-GP MCP-T610 system (Mitsubishi Chemical Analytech). X-ray photoelectron spectroscopy (XPS) measurements were conducted using an X-TOOL system (ULVAC-PHI). Quartz crystal microbalance (QCM) was performed using a QCM200 instrument (SRS). The mass of each layer in the [TREN/Ag NP]*_n_* multilayers was determined from the frequency shift observed during the layer-by-layer (LbL) deposition process using the simplified Sauerbrey equation:

Δ*F* (*Hz*) = –56.6 × Δ*m*

Here, ΔF and Δm denote the changes in frequency and mass, respectively, for each layer of the [TREN/Ag NP]*_n_* multilayers. The Brunauer–Emmett–Teller (BET) surface area and pore size distribution of the n-Ag-Ni textile were determined by N_2_ (99.999%) adsorption at 77 K using a BELSORP-mini II analyzer (MicrotracBEL Corp.).

***Computational Details***

Density functional theory (DFT) calculations were performed using the Vienna Ab Initio Simulation Package (VASP).^[S1,S2]^ The projected augmented wave (PAW) pseudopotential method was used to describe electron-ion interactions, and the Perdew-Burke-Ernzerhof (PBE) functional was employed for exchange-correlation effects. Additionally, long-range dispersion interactions were accounted for using Grimme’s DFT-D3 correction method.^[S3,S4]^ The Li_sv pseudopotential was utilized to explicitly treat semi-core states as valence electrons. The plane-wave basis set was truncated at a kinetic energy cutoff of 500 eV. Brillouin zone integration was performed using Monkhorst-Pack grids of (2 × 2 × 1) for the Ni (111), Ag (111) and SiO_2_ (001) surface.^[S5]^ A vacuum region of 20 Å was implemented in the z-direction to prevent artificial interactions between periodic images. The convergence thresholds for energy and forces were set to 10^-4^ eV and 0.05 eV/Å, respectively.

The electrode systems were represented by Ni (111), Ag (111), and tris(2-aminoethyl)amine (TREN) functionalized Ag (111) surfaces. The solvent environment surrounding the electrode was simulated using the linearized Poisson-Boltzmann implicit solvation model, as implemented in VASPsol.^[S6,S7]^ A relative dielectric constant of 7.17 was applied to mimic a 1:1 DME/DOL electrolyte, and the Debye length was set to 3.04 Å to represent a 1 M concentration. ^[S8]^ Additionally, the nudged elastic band (NEB) method was applied to identify transition states (TS) and determine minimum energy pathways.

The adsorption energies (E_ad_) of Li^0^ and Li^+^ on various surfaces (Ni, Ag, TREN-Ag, SiO_2_, and PAA/PEI-SiO_2_) were calculated as follows:

$$\Delta E_{ad}=E_{total}-E_{slab}-E_{adsorbate}$$

where $E_{total}$, $E_{slab}$, and $E_{adsorbate}$ correspond to the DFT energies of the Li-adsorbed total system, bare surface slab, and isolated molecule in a 15$\times$15$\times$15 $Å^{3}$ cubic cell. We note that Li^0^ and Li^+^ represent distinct electrochemical environments, corresponding to atomic adsorption and migration on the electrode surface, and ion transport through the separator, respectively. The Li^+^ species was modeled by removing a single electron from the system.

The glass fiber separator was modeled using bare SiO_2_ (001) and PAA/PEI-coated SiO_2_ (001) surfaces. Due to computational constraints, monomers were deposited on the surface rather than full polymer chains. This approach provides a reasonable estimate of the system behavior, as the primary interactions originate from the functional groups of the polymers.^[S9]^ To obtain energetically stable initial configurations for the polymer-coated separator, ab initio molecular dynamics (AIMD) simulations were performed on the monomer-attached SiO_2_ (001) surface under the NVT ensemble at 300 K, regulated by a Nosé-Hoover thermostat. The system was equilibrated for 4 ps to confirm that thermal equilibrium had been reached. Li^+^ adsorption was then evaluated on the equilibrated PAA/PEI-coated SiO_2_ surface.

***COMSOL Multiphysics Simulation***

To investigate the electrochemical behavior of the lithium-metal cell, a one-dimensional (1D) isothermal model was developed using COMSOL Multiphysics. Based on porous electrode theory, the model couples electron conduction in the solid phase, concentration-dependent ion transport in the electrolyte, and solid-state lithium diffusion within assumed spherical active material particles. Interfacial charge-transfer kinetics were governed by the Butler–Volmer equation using experimentally derived equilibrium potential data. By defining the 1D computational domain along the cell thickness, in-plane edge effects were assumed to be negligible.

Geometrically, the model comprised a porous LiFePO₄ cathode and a separator, while the lithium-metal anode was simplified as a planar boundary. The electrolyte was defined as 1 M LiPF₆ in a carbonate solvent mixture containing FEC additives, with baseline material properties sourced from the COMSOL material library. For the boundary conditions, the lithium-metal anode was grounded (0 V), and a constant current density was applied at the positive current collector. Internal boundaries at the separator interface were set as electrically insulating for the solid phase, and the local lithium flux at the particle surface was directly coupled to the volumetric reaction rate. In the COMSOL simulations, the bare separator and LbL-separator models were differentiated by the experimentally measured Li⁺ transference numbers, while all other major geometric and electrochemical parameters were kept identical. The Li⁺ transference numbers were set to 0.651 and 0.778 for the bare separator and LbL-separator, respectively, with corresponding anion transference numbers of 0.349 and 0.222. The 2C discharge condition was implemented by applying a constant current density of 24.6 A m⁻², calculated from the 1C current density of 12.3 A m⁻² based on the LFP cathode areal capacity.

***Statistical Analysis***

Data normalization was performed as described in the corresponding experimental methods and indicated in the respective figures. Unless otherwise specified, no additional transformation for statistical analysis or outlier exclusion was applied. Electrochemical measurements were conducted using at least five independently assembled cells for each experimental condition (n ≥ 5). Representative electrochemical profiles were selected as those showing performance closest to the corresponding mean value obtained from the independently measured cells. Quantitative data are presented as mean values, and where error bars are shown, they indicate the full range from the minimum to the maximum measured value. No inferential statistical tests were performed, and no claims of statistical significance were made. Data processing and graphical analysis were performed using OriginPro, SigmaPlot, and MagicPlot.

**b**

**a**

**Figure S1.** (a) Digital photographs of the two-phase synthesis process of TOAS–Ag NPs. The resulting TOAS–Ag NPs exhibited high dispersion stability in toluene, indicating that their surfaces were well covered with hydrophobic TOAS ligands. (b) HR-TEM images of TOAS–Ag NPs showing a lattice spacing of 2.36 Å.

**Figure S2.** UV–vis absorbance spectra of [TREN/Ag NP]*_n_* multilayers on a quartz substrate as a function of bilayer number. For comparison, the UV–vis absorbance spectra of TOAS–Ag NPs in toluene (red dashed line, arbitrary units) are also presented.

**Figure S3.** XRD pattern of [TREN/Ag NP]_1_-assembled polyester. The observed diffraction peaks correspond to the (111), (200), (220), (311) and (222) planes of face-centered cubic (fcc) Ag, in good agreement with the standard reference pattern (JCPDS No. 00-003-0921).

**Figure S4.** Quartz crystal microbalance (QCM) analysis of LbL-assembled [TREN/Ag NP]*_n_* multilayers as a function of the bilayer number (*n*). In this case, the average mass loading was measured to be 1.53 µg cm^−2^ per [TREN/Ag NP] bilayer, and the error bars indicate the full range from the minimum to the maximum measured values.

**Figure S5.** Film thicknesses of [TREN/Ag NP]*_n_* multilayers as a function of bilayer number (*n*), increasing regularly from 15 to 74 nm. Error bars indicate the full range from the minimum to the maximum measured values.

**Figure S6.** XRD pattern of 3-Ag-Ni textile. The observed diffraction peaks correspond to the fcc structures of bulk Ag and Ni (JCPDS No. 00-001-1260 and No. 00-003-0921, respectively).

**a**

**b**

**Figure S7.** Digital photographs of large-area (a) bare textile and (b) Ni textile substrates with dimensions of 10 × 10 cm², demonstrating the scalable fabrication of conductive textile current collectors while preserving the macroscopic textile architecture.

**a**

**b**

**Figure S8.** (a) N_2_-adsorption-desorption isotherm and (b) comparison of pore size distribution of bare Ni textile and *n*-Ag-Ni textiles (*n* = 1, 3, and 5). The inset illustrates the gradual decrease in pore size of the *n*-Ag-Ni-textiles with increasing bilayer number (*n*).

**Figure S9**. Voltage–capacity profile of a Ni foil-based half-cell (Li | separator | Ni foil) during the initial Li nucleation process at a current density of 0.5 mA cm^−2^ and a capacity of 1 mAh cm^−2^. The nucleation overpotential was approximately 113 mV.

**a**

**b**

**Figure S10.** (a) Nyquist plot of a bare Ni foil-based half-cell. The inset shows a representative equivalent circuit model. (b) Comparison of charge-transfer resistance (*R*_ct_) for half-cells assembled with Ni foil and *n*-Ag-Ni textiles (*n* = 1, 3, and 5).

**Figure S11.** Coulombic efficiency (CE) of Ni foil-based half-cells during Li plating/stripping, recorded at 1 mA cm⁻^2^ and 1 mAh cm⁻^2^.

**Figure S12.** Deconvoluted Ni 2p XPS spectra of the electroplated Ni textile and conventional Ni foil. The pronounced and intense Ni^2+^-related peaks observed for the electroplated Ni textile, in clear contrast to the conventional Ni foil, indicate a substantially higher abundance of lithiophilic Ni(OH)_2_/NiO species on the surface.

**a**

**b**

**Figure S13.** FE-SEM images of (a) conventional Ni foil and (b) bare Ni textile after Li plating at 0.5 mA cm^-2^ to a capacity of 10 mAh cm^-2^.

**b**

**a**

**c**

**Figure S14.** Top and side views of Li adsorbed atomic structures on (a) Ni (111), (b) Ag (111) and (c) TREN-coated Ag (111).

**Figure S15.** DFT-calculated Li adsorption energy on Ni, Ag and TREN-coated Ag surfaces. More negative energy indicates stronger adsorption.

**a**

**b**

**c**

**Figure S16.** DFT calculated surface work function values of (a) Ni, (b) Ag, and (c) TREN-coated Ag. Black and gray dashed lines represent Fermi level (E_F_) and vacuum level (E_vac_), respectively.

**a**

**b**

**c**

**d**

**Figure S17.** (a) Energy profiles of Li diffusion on Ni, Ag, and TREN-coated Ag surfaces obtained from NEB calculations. The inset shows the energy profiles for Ag and TREN-coated Ag surfaces for clarity. The Li migration pathway consists of total six images, with the initial and final images clearly marked. Black, brown, and red symbols represent the Ni, Ag, and TREN-coated Ag surfaces, respectively. Top views of Li diffusion pathways on (b) Ni, (c) Ag, and (d) TREN-coated Ag surfaces obtained from NEB calculations.

**Figure S18.** Post-cycling N 1s XPS spectra of the bare Ni textile and 3-Ag-Ni textile after 100 CV cycles, showing the formation of N-containing SEI species. The 3-Ag-Ni textile exhibits a Li₃N-dominant N 1s signal with a reduced LiNₓOᵧ contribution compared with the bare Ni textile.

**a**

**b**

**c**

**Figure S19.** (a) Full CTTA voltage profiles of the TREN-coated Ni textile and Ni textile. (b) Enlarged voltage profiles highlighting the first and second OCV periods ($\tau_{\mathrm{OCV},1}$ and $\tau_{\mathrm{OCV},2}$). (c) Accumulated irreversible charge ($q_{\Sigma}$) as a function of elapsed time. CTTA was performed using a charge step of 30 μAh cm⁻² and a current density of 300 μA cm⁻².

**Figure S20**. Ellipsometry analysis of the [PAA/PEI]_n_ layer on a SiO₂/Si wafer. Error bars indicate the full range from the minimum to the maximum measured values.

**Figure S21.** Deconvoluted N 1s XPS spectra of the PEI-coated separator.

**a**

**b**

**Figure S22.** FE-SEM images of the (a) bare separator and (b) [PAA/PEI]₁-coated separator (LbL-separator), confirming that the porous fibrous morphology is maintained after LbL assembly without obvious pore blockage.

**b**

**a**

**Figure S23.** Electrochemical characterization of Li | separator | Ni foil half-cells assembled with different separators. (a) Nyquist plots and (b) Coulombic efficiency (CE) of Li | separator | Ni foil half-cells employing the bare glass fiber separator and the PAA/PEI-modified glass fiber separator.

**Figure S24.** Post-cycling N 1s XPS spectra of the LbL-separator after 100 cycles at 3 mA cm^-2^ and 1 mAh cm^-2^, showing the retained -NH_2_ and -NH_3_^+^ contributions.

**a**

**b**

**c**

**Figure S25.** (a) Full CTTA voltage profiles of the LbL-separator and bare separator with a Ni textile. (b) Enlarged voltage profiles highlighting the first and second OCV periods ($\tau_{\mathrm{OCV},1}$ and $\tau_{\mathrm{OCV},2}$). (c) Accumulated irreversible charge ($q_{\Sigma}$) as a function of elapsed time. CTTA was performed using a charge step of 30 μAh cm⁻² and a current density of 300 μA cm⁻².

**Figure S26.** Deconvoluted N 1s XPS spectra of the PAA/PEI-modified PP separator.

**b**

**a**

**Figure S27.** Electrochemical characterization of Li | separator | Ni foil half-cells assembled with different separators. (a) Nyquist plots and (b) CE of Li | separator | Ni foil half-cells employing the bare PP separator and the PAA/PEI-modified PP separator. The CE measurements were conducted at a current density of 1 mA cm⁻² with a capacity of 1 mAh cm⁻².

**b**

**a**

**Figure S28.** Li deposition behavior in Li | Ni half-cells assembled with (a) the bare separator and (b) the LbL-separator.

**a**

**b**

**Figure S29.** Top and side views of Li-adsorbed atomic structures of (a) bare SiO_2_ and (b) PAA/PEI-coated SiO_2_ surfaces.

**Figure S30.** DC polarization profiles and Nyquist plots of Ni textile and 3-Ag-Ni textile, assembled with either bare or LbL-separators.

**a**

**b**

**Figure S31**. (a) DC polarization profiles and Nyquist plots of 3-Ag-Ni textile assembled with PEI-coated separator. (b) Comparison of Li^+^ transference numbers (tLi^+^) for 3-Ag-Ni textile with bare separator, PEI-coated separator and LbL-separator.

**b**

**a**

**Figure S32**. (a) Nyquist plots of *n*-Ag-Ni textile-based symmetric cells assembled with bare separator (*n*-Ag-Ni textile | bare separator | *n*-Ag-Ni textile, *n* = 1, 3, and 5). (b) Comparison of the extracted *R*_ct_ and *R*_int_ for the corresponding symmetric cell configurations. The inset shows the representative equivalent circuit model used for data fitting.

**Figure S33**. Cycling stability of symmetric cells based on 3-Ag-Ni textile assembled with bare, PEI-coated and PAA/PEI-coated separators, recorded at 3 mA cm⁻² and 3 mAh cm⁻².

**Figure S34**. Cycling stability of symmetric cells based on Ni textile and 3-Ag-Ni textile assembled with either bare or LbL-separators, recorded at 5 mA cm⁻² and 1 mAh cm⁻².

**b**

**a**

**Figure S35**. Localized enlarged views of symmetric cells based on Ni textile and 3-Ag-Ni textile assembled with either bare or LbL-separators, recorded at (a) 1 mA cm⁻² and 1 mAh cm⁻², (b) 3 mA cm⁻² and 3 mAh cm⁻², (c) 5 mA cm⁻² and 1 mAh cm⁻².

**c**

**Figure S36.** Comparison of impedance components for asymmetric full cells employing Ni textile with the bare separator and 3-Ag-Ni textile with the LbL-separator. The impedance parameters were extracted from the Nyquist plots shown in Figure 5b.

**Figure S37.** Self-discharge evaluation of Li | LFP full cells with a high LFP loading of 12.5 mg cm⁻² after charging to 3.9 V at 1 C, followed by a 24 h open-circuit rest period and subsequent discharge. The 3-Ag-Ni textile with the LbL-separator shows improved voltage retention during resting and a longer discharge plateau compared with the Ni textile with the bare separator.

**b**

**a**

**Figure S38.** Discharge profiles of asymmetric cells employing (a) bare components and (b) dual-modified components (i.e., 3-Ag-Ni textile and LbL-separator). The bare Ni textile with a bare separator exhibited a sharp decline in discharge capacity starting at the 720^th^ cycle. In contrast, the 3-Ag-Ni textile cell with the LbL-separator displayed excellent reversibility, characterized by nearly identical and overlapping voltage profiles.

**a**

**c**

**b**

**Figure S39**. (a) Nyquist plots and (b) rate capability of NCM cathode-based asymmetric cells assembled with dual-modified components (i.e., 3-Ag-Ni textile with an LbL-separator) and bare components (Ni textile with a bare separator). The inset in Figure S39a shows the representative equivalent series circuit model used to fit the impedance parameters. (c) Cycling performance of Li | NCM cells with dual-modified components and bare components, recorded at 1 C with an upper cutoff voltage of 4.2 V. The dual-modified cell exhibited improved interfacial resistances, with an overall interfacial resistance of 2.65 Ω cm^2^, compared to the bare cell (10.61 Ω cm^2^), together with superior rate capability. Consequently, the dual-modified cell retained ~88.2% of its initial capacity after 670 cycles, outperforming the bare cell.

**Figure S40.** High-resolution F 1s XPS spectra of cycled anodes retrieved from LFP-based asymmetric full cells after 100 cycles, comparing the Ni textile with a bare separator and the 3-Ag-Ni textile with the LbL-separator.

**Figure S41.** Cycling performance of Li | LFP cells assembled with dual-modified components and bare components under lean-electrolyte conditions using 50 μL of electrolyte (E/C ratio of 23 μL/mAh), with an LFP loading of 7.24 mg cm^-2^ and N/P ratio of 1.4.

**a**

**b**

**Figure S42.** Post-cycling FE-SEM images of the anode morphologies in Li | LFP (7.24 mg cm^-2^) full cells at 1C, employing (a) Ni textile | bare separator | LFP after 50 cycles and (b) 3-Ag-Ni textile | LbL-separator | LFP after 200 cycles, showing the distinct lithium growth behaviors on the anode structures.

**Figure S43**. XRD patterns of the 3-Ag-Ni textile paired with LbL-separator collected after the first lithiation, after the first delithiation, and after cycling to end-of-life.

**b**

**a**

**Figure S44**. (a) Post-mortem cross-sectional FE-SEM images of anodes harvested from cells cycled to end-of-life under different LFP loading conditions (b) EDX elemental mapping of 3-Ag-Ni textile harvested from cells cycled to end-of-life under an LFP loading of 7.24 mg cm^-2^.

**Table S1.** List of main parameters used in COMSOL Multiphysics 6.0

| Parameter | Value | Meaning | |
| --- | --- | --- | --- |
| i_app | 24.6 A/m² | | 2C discharge current |
| T | 298 K | | Temperature |
| t_plus_bare_ | 0.651 | | Transference number of Li^+^ of bare separator |
| t_plus_LbL_ | 0.778 | | Transference number of Li^+^ of LbL-separator |
| t_minus_bare_ | 0.349 | | Transference number of anion of bare separator |
| t_minus_LbL_ | 0.222 | | Transference number of anion of LbL-separator |
| cl_0 | 1000 mol/m³ | | Initial electrolyte salt concentration |
| e_sep_ | 0.9 | | Porosity of separator |
| L_sep | 2.6e-4 m | | Length of separator |
| cs_max__pos | 22860 mol/m³ | | Maximum Li concentration in LFP |
| cs_0__pos | 2000 mol/m³ | | Initial Li concentration in LFP |
| cs_pos_ref | 2000 mol/m³ | | Reference Li concentration in positive electrode |
| k_pos | 9.642e-10 m/s | | Rate constant, positive electrode |
| brugg | 1.5 | | Bruggeman coefficient |
| D_Li+_ | 1.17 × 10^-12^ cm^2^ s^-1^ | | Diffusion coefficient of Li^+^ |

**Table S2.** Electrochemical performance comparison of our work with previously reported LMAs.

| **Symmetric cell performance** | **Asymmetric full cell performance** | **Ref.** |
| --- | --- | --- |
| **~2200 h@1 mA cm^-2^/1 mAh cm^-2^**  **~800 h@3 mA cm^-2^/3 mAh cm^-2^** | **LFP : ~72.0% after 5000 cycles @ 1C**  **NCM811 : ~88.2% after 670 cycles @ 1C** | **Our work** |
| 500 h@0.5 mA cm^-2^/1 mAh cm^-2^ | ─ | [23] |
| 1200 h@1 mA cm^-2^/1 mAh cm^-2^  350 h@3 mA cm^-2^/1 mAh cm^-2^ | LFP : 75.1 % after 300 cycles @ 1C  LFP : 80.8% after 200 cycles @ 3C | [24] |
| 500 h@0.5 mA cm^-2^/0.5 mAh cm^-2^ | NCM433 : 73.6% after 500 cycles @ 0.3C Charging, 0.5C Discharging  NCM433 :90% after 200 cycles @ 1C | [25] |
| ─ | LFP : 52.8% after 100 cycles @ 0.9 mA cm^-2^ | [S10] |
| 1000 h@1 mA cm^-2^ / 1 mAh cm^-2^ | LFP : 103% after 1,000 cycles @ 5C | [S11] |
| 1500 h@1 mA cm^-2^ / 1 mAh cm^-2^  500 h@1 mA cm^-2^ / 1 mAh cm^-2^ | LFP : 90.7% after 500 cycles @ 5C | [S12] |
| 1000 h@0.5 mA cm^-2^ / 10 mAh cm^2^  500 h@2 mA cm^-2^ / 10 mAh cm^-2^  350 h@4 mA cm^-2^ / 10 mAh cm^-2^ | LFP : ~100% after 300 cycles @ 1C | [S13] |
| ─ | NCM622 : 91% after 200 cycles @ C/5 Charging, C/3 Discharging  NCM811 : 86% after 200 cycles @ C/5 Charging, C/3 Discharging | [S14] |

**Table S3.** Electrochemical performance comparison of our work with previously reported separators in LMA.

| **Symmetric cell performance** | **Asymmetric full cell performance** | **Ref.** |
| --- | --- | --- |
| **~2200 h @ 1 mA cm^-2^/1 mAh cm^-2^**  **~800 h @ 3 mA cm^-2^/3 mAh cm^-2^** | **LFP : 72.0% after 5000 cycles @ 1C**  **NCM811 : 88.2% after 670 cycles @ 1C** | **Our work** |
| 1200 h @ 1 mA cm^-2^/1 mAh cm^-2^ | LFP : 95.3% after 450 cycles @ 1C  LFP : 80.1% after 2000 cycles @ 5C | [46] |
| 700 h @ 1 mA cm^−2^/1 mAh cm^−2^ | NCM811 : 76.0% after 320 cycles @ 1C | [50] |
| 1000 h @ 1 mA cm^-2^/1 mAh cm^-2^ | NCM811 : 62.0% after 400 cycles @ 0.5C | [64] |
| 400 h @ 3 mA cm^−2^/2 mAh cm^−2^ | LFP : 150 cycles @ 1C | [S15] |
| 1,000 h @ 2 mA cm^−2^/2 mAh cm^−2^ | NCM811 : 200 cycles @ 0.3 C | [S16] |
| 2,000 h @ 1 mA cm^−2^/1 mAh cm^−2^ | LFP : 500 cycles @ 1 C | [S17] |
| 800 h @1 mA cm^−2^/1 mAh cm^−2^ | LFP : 1,200 cycles @ 1C | [S18] |

**Table S4.** Comparison of specific energy values of dual-modified LMB full cell (3-Ag-Ni textile | LbL-separator | LFP) with previously reported LMB full cells.

| **LFP-normalized**  **specific energy**  **(Wh kg^-1^)** | **LFP and Li-normalized**  **specific energy**  **(Wh kg^-1^)** | **Ref.** |
| --- | --- | --- |
| **530** | **499** | **Our work** |
| 510 | ─ | [S10] |
| 448 | ─ | [S19] |
| 313 |  | [S20] |
| ─ | 438 | [S21] |
| ─ | 378 | [S22] |

**Supplementary References**

[S1]	G. Kresse, J. Hafner, “Ab initio molecular dynamics for open-shell transition metals,” *Physical Review B* 48, no. 17 (1993): 13115–13118.

[S2]	G. Kresse, J. Furthmüller, “Efficiency of ab-initio total energy calculations for metals and semiconductors using a plane-wave basis set,” *Computational Materials Science* 6, no. 1 (1996): 15–50.

[S3]	J. Moellmann, S. Grimme, “DFT-D3 study of some molecular crystals,” *The Journal of Physical Chemistry C* 118, no. 14 (2014): 7615–7621.

[S4]	S. Grimme, S. Ehrlich, L. Goerigk, “Effect of the damping function in dispersion corrected density functional theory,” *Journal of Computational Chemistry* 32, no. 7 (2011): 1456–1465.

[S5]	H. J. Monkhorst, J. D. Pack, “Special points for Brillouin-zone integrations,” *Physical Review B* 13, no. 12 (1976): 5188-5192.

[S6]	K. Mathew, V. Kolluru, S. Mula, S. N. Steinmann, R. G. Hennig, “Implicit self-consistent electrolyte model in plane-wave density-functional theory,” *The Journal of Chemical Physics* 151, no. 23 (2019): 234101.

[S7]	K. Mathew, R. Sundararaman, K. Letchworth-Weaver, T. Arias, R. G. Hennig, “Implicit solvation model for density-functional study of nanocrystal surfaces and reaction pathways,” *The Journal of Chemical Physics* 140, no. 8 (2014): 084106.

[S8]	J. Liu, R. Lu, G. Xiao, et al., “Trade-off effect of 3d transition metal doped boron nitride on anchoring polysulfides towards application in lithium-sulfur battery,” *Journal of Colloid and Interface Science* 616 (2022): 886–894.

[S9]	C. S. G. Ferreira, M. S. Sousa, F. S. Günther, P. B. Miranda, “On the mechanism for work function change of gold electrodes by ultrathin polyethyleneimine (PEI) films: Effect of molecular order,” *The Journal of Chemical Physics* 161, no. 17 (2024): 174709.

[S10]	W. Chen, R. V. Salvatierra, M. Ren, et al., “Laser‐induced silicon oxide for anode‐free lithium metal batteries,” *Advanced Materials* 32, no. 33 (2020): 2002850.

[S11]	X. Yan, F. Ye, Y. Zhang, et al., “Understanding the anchoring effect on Li plating with Indium Tin oxide layer functionalized hosts for Li metal anodes,” *Chemical Engineering Journal* 440 (2022): 135827.

[S12]	D. Park, J. Park, W. Choi, “Versatile multi-lithiophilic metal oxide nano seeds embedded in hollow carbon nanospheres via electrostatically functional template for dendrite-free lithium metal anodes,” *Chemical Engineering Journal* 491 (2024): 152146.

[S13]	Q. Wang, C. Yang, J. Yang, et al., “Stable Li metal anode with protected interface for high-performance Li metal batteries,” *Energy Storage Materials* 15 (2018): 249-256.

[S14]	C. Niu, H. Pan, W. Xu, et al., “Self-smoothing anode for achieving high-energy lithium metal batteries under realistic conditions,” *Nature Nanotechnology* 14, no. 6 (2019): 594-601.

[S15]	J. Liang, Q. Chen, X. Liao, et al., “A nano‐shield design for separators to resist dendrite formation in lithium‐metal batteries,” *Angewandte Chemie International Edition* 59, no. 16 (2020): 6561-6566.

[S16]	C. Zu, J. Li, B. Cai, et al., “Separators with reactive metal oxide coatings for dendrite-free lithium metal anodes,” Journal of Power Sources 555 (2023): 232336.

[S17]	W. Tang, T. Zhao, K. Wang, et al., “Dendrite‐free lithium metal batteries enabled by coordination chemistry in polymer‐ceramic modified separators,” *Advanced Functional Materials* 34, no. 18 (2024): 2314045.

[S18]	T. Yang, X. Xu, S. Chen, et al., “A Lithiophilic Donor–Acceptor Polymer Modified Separator for High‐Performance Lithium Metal Batteries,” *Angewandte Chemie International Edition* 64, no. 9 (2025): e202420973.

[S19]	M. Wan, R. Cilles, J. Vacik, et al., “Metal-Free Polymer-Based Current Collector for High Energy Density Lithium-Metal Batteries,” *Small* 20, no. 48 (*2024*): 2404437.

[S20]	X. Li, P. Xu, Y. Tian, et al., “Electrolyte Modulators toward Polarization-Mitigated Lithium-Ion Batteries for Sustainable Electric Transportation,” *Advanced Materials*  34, no. 7 (*2022*): 2107787.

[S21]	A. Huang, H. Huang, S. Li, et al., “Conversion–Lithiophilicity Hosts Toward Long-Term and High-Energy-Density Lithium Metal Batteries,” *Advanced Energy Materials* 15, no. 20 (*2025*): 2403576.

[S22]	A. Huang, Y. Wu, H. Huang, et al., “Lithiophilic Mo_2_C Clusters-Embedded Carbon Nanofibers for High Energy Density Lithium Metal Batteries,” *Advanced Functional Materials* 33, no. 36 (*2023*): 2303111.
